# Slide‐Crosslinked Polyrotaxane Topological Networks: Quasi‐Solid Electrolyte for High‐Voltage Lithium Metal Batteries

**DOI:** 10.1002/advs.202508598

**Published:** 2025-07-17

**Authors:** Huirong Zhu, Xiaoyue Zeng, Xuewei Liu, Jiaxing Zhu, Jinghao Hua, Jinle Lan, Yunhua Yu, Xiaoping Yang

**Affiliations:** ^1^ State Key Laboratory of Organic‐Inorganic Composites College of Materials Science and Engineering Beijing University of Chemical Technology North Third Ring Road 15, Chaoyang Beijing 100029 P. R. China

**Keywords:** high‐voltage lithium metal batteries, in‐situ preparation, slide‐crosslinked, stable interfaces

## Abstract

Although polymer‐based electrolytes offer advantages like low cost, favorable interfacial compatibility, and processability for solid‐state lithium metal batteries with high safety and high energy density, conventional linear polymer‐based electrolytes suffer from inadequate oxidation resistance and mechanical strength at operating voltages above 4.5 V, causing rapid capacity degradation and reduced battery lifespan. Inspired by the mechanical slide‐ring structure of polyrotaxanes (PR), a series of high‐voltage‐resistant sliding crosslinked quasi‐solid electrolytes (PMBA‐PPR_x_) is designed and synthesized via in situ thermal polymerization of varying amounts of vinyl functional polyrotaxanes (PPRs) with N,N'‐methylenebisacrylamide (MBA). The optimal PMBA‐PPR_5_ electrolyte realizes the synergistic enhancement of both mechanical properties and high‐voltage‐resistant electrochemical properties as well as the good interfacial compatibility. The dynamic slide ring structure of PPRs effectively dissipates the energy generated by lithium dendrite growth, thereby maintaining the mechanical robustness of the electrolyte during battery cycling and achieving lithium deposition/stripping behavior for more than 2000 h at 0.5 mA cm^−2^. The strong polar amide groups of MBA not only improve the lithium‐ion transference number (0.69), but also enhance the high‐voltage stability of the electrolyte (∼ 5.5 V), ultimately resulting in excellent cycling stability and capacity retention of Li|PMBA‐PPR_5_|LFP and Li|PMBA‐PPR_5_|NCM811 cells. This slide‐crosslinked polyrotaxane topological dynamic structure provides a new strategy for the design of high‐voltage lithium metal electrolytes.

## Graphical abstract

A series of sliding crosslinked quasi‐solid electrolyte (PMBA‐PPR_x_) is designed for high‐voltage lithium metal batteries, concurrently addressing the critical bottlenecks of conventional polymer electrolytes: restricted high‐voltage tolerance (< 4.5 V vs. Li/Li⁺) and unstable interfacial compatibility. The optimal slide‐crosslinked electrolyte (PMBA‐PPR_5_) effectively dissipates the stress generated by lithium dendrite growth and adjusts a homogeneous Li^+^ flux distribution, enabling lithium metal batteries with stable cyclability and high rate‐capability.

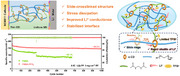

## 1 Introduction

The rapid expansion of the electric vehicle market has spurred increasing demand for batteries with high energy and power density, long cycle‐life, and safety.^[^ ^1^ ^]^ Lithium metal is currently considered the best material to replace traditional graphite anodes due to its ultrahigh specific capacity (3860 mAh g^−1^) and low redox potential (−3.04 V vs standard hydrogen electrode).^[^ ^2^ ^]^ However, the liquid electrolyte used in lithium‐metal batteries not only fails to inhibit the growth of lithium dendrites during the cycling process, posing a safety risk of thermal runaway or even explosion of the battery, but also limits its compatibility with high‐voltage cathodes due to uncontrollable oxidative decomposition by side reactions.^[^ ^3^ ^]^ Solid‐state batteries, recognized for their superior mechanical stability and safety, are positioned as an essential technology to substantially increase battery energy density in next‐generation energy storage solutions.^[^ ^4^ ^]^

Solid polymer electrolytes (SPEs) are considered one of the most promising solid‐state electrolyte systems to be commercialized first due to its strong designability, simple processing and low cost.^[^ ^5^, ^6^ ^]^ Currently, the mainstream polymer electrolyte systems, primarily composed of linear polymer matrices such as poly (ethylene oxide) (PEO) and polyacrylonitrile (PAN), exhibit insufficient mechanical properties to effectively suppress the growth of lithium dendrites.^[^ ^7^ ^]^ Meanwhile, the inherently limited electrochemical stability window of linear polymer electrolytes severely reduces their compatibility with high‐voltage cathode materials and consequently impedes energy density enhancement.^[^ ^8^ ^]^ The crucial issues in the development of polymer solid‐state electrolytes have been addressed by advanced structural design strategies.^[^ ^9^ ^]^ Wang et al synthesized a novel triblock copolymer and blended it with a long‐chain poly (vinylidene fluoride‐hexafluoropropylene) (PVFH) to obtain a new high‐voltage polymer electrolyte suitable for lithium metal batteries. The highly entangled structure was constructed by the strong interaction between polymer segments, which effectively enhanced the mechanical properties of the electrolyte (the tensile strength reaches 19.83 MPa).^[^ ^10^ ^]^ Motivated by the synergistic effect of polar groups including nitrile, ester, and anhydride groups, Min et al achieved controlled copolymerization of ethyl cyanoacrylate (ECA)‐based electrolytes. The electrolyte system possessed extremely high ionic conductivity and improved mechanical robustness.^[^ ^11^ ^]^ Clearly, the polymer cross‐linking network constructed by chemical or physical interactions through molecular level design can effectively improve the mechanical stability of the system and form stable ionic conduction pathways, achieving the synergistic enhancement of the mechanical and electrochemical properties of the polymer electrolyte.^[^ ^12^, ^13^, ^14^, ^15^ ^]^

As a topological supramolecule with mechanical interlocking structure, cyclodextrin‐based polyrotaxane is a kind of “pulley” structure that is obtained by self‐assembly of multiple cyclodextrin molecules to long‐chain molecules through host–guest effect.^[^ ^16^, ^17^, ^18^ ^]^ Under the condition of external force stimulation, cyclodextrins can effectively relieve the stress concentration and improve the mechanical stability of the materials through reciprocal slide and rotation on the axis.^[^ ^19^ ^]^ Concurrently, the abundant hydroxyl groups inherent in cyclodextrins provide a versatile platform for the functionalization of polyrotaxanes, enabling their tailored integration into energy material systems.^[^ ^20^, ^21^ ^]^ Yan et al. designed and synthesized a crosslinker containing a highly dynamic boronate anion to further obtain a cyclodextrin‐based polyrotaxane (PRX) network, which integrated the unique sliding structure and stabled cycling behavior.^[^ ^22^ ^]^ Ding et al. developed a sliding crosslinked gel polymer electrolyte containing strong polar groups of isocyanates for lithium‐metal batteries, which verifies the great potential of polyrotaxane materials for lithium‐metal batteries, and provides a new strategy for the design of polymer electrolytes.^[^ ^23^ ^]^

**Figure 1:**
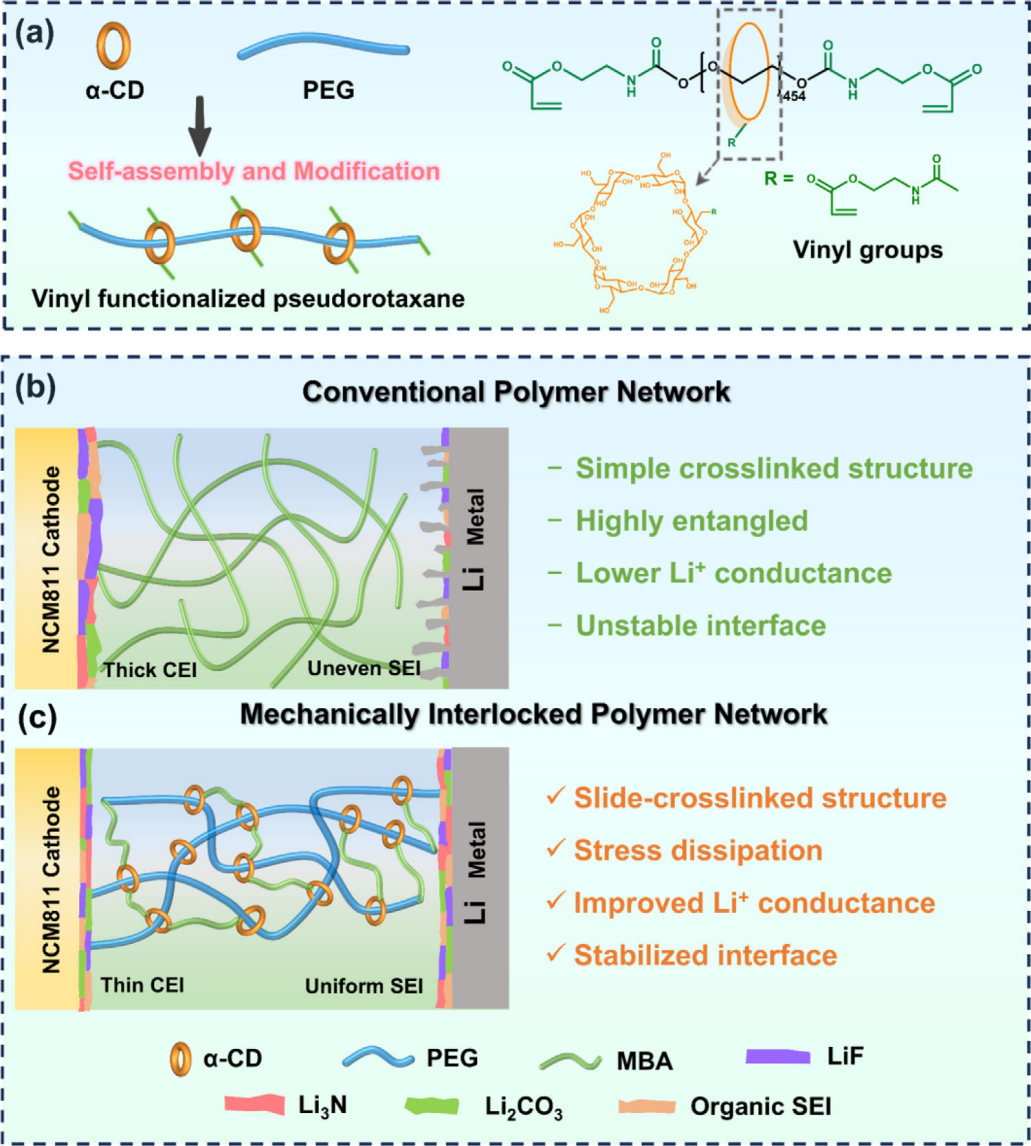
a) Preparation of vinyl functionalized pseudorotaxane (PPRs). b) Conventional crosslinked polymer network. c) Mechanically interlocked polymer network (PMBA‐PPR_x_).

Herein, we engineered a high‐voltage‐resistant quasi‐solid topological dynamic structures based on vinyl functional polyrotaxanes (PPRs) by in situ polymerization process (**Figure** **1**a). The PPRs were synthesized through a high efficiency and high yield two‐step method (Figure S1, Supporting Information), in which a special host–guest effect of cyclodextrin (α‐CD) molecules was utilized to self‐assemble with long‐chain polyethylene glycol (PEG) molecules, obtaining a dynamic mechanical structure like molecular pulleys.^[^ ^24^, ^25^ ^]^ Additionally, we selected N,N'‐methylenebisacrylamide (MBA) as the crosslinker containing polar amide groups to construct a dynamic crosslinked framework with PPRs. This mechanically interlocked polymer network effectively dissipates the stress concentrated during the lithium plating/stripping process, and dynamically adapts to the volumetric changes of the lithium metal anode, enabling the lithium symmetric batteries to be stably cycled under 0.5 mA cm^−2^ for 2000 h. Meanwhile, the CD molecules synergistically coordinate with Li^+^, where the dynamic motion of CD molecules facilitates rapid ion transport, thereby improving the electrochemical performance of the electrolyte, and the room‐temperature ionic conductivity reaches 3.08 × 10^−3^ S cm^−1^. Additionally, the structure restricts the movements of the TFSI^−^ anions, achieving the Li^+^ transference number of 0.69.^[^ ^26^ ^]^ Consequently, the Li||NCM811 full cell can be stably cycled for 300 cycles at 0.5 C. This slide‐crosslinked PPR‐based polymer electrolyte demonstrates a great potential for the compatibility of high‐voltage cathodes.

## 2 Results and Discussion

### 2.1 Design and Preparation of PMBA‐PPR_x_ Electrolytes

Typically, conventional crosslinked polymer networks (Figure 1b) form stable 3D networks via chemical or physical crosslinking, ^[^ ^27^ ^]^ exhibiting enhanced mechanical strength and thermal stability. However, their electrochemical performance is often compromised by the restrictions on the movement of chain segments. Additionally, entanglement between chain segments is easy to occur with the increase of molecular weight of the crosslinked polymer network.^[^ ^28^, ^29^ ^]^ In contrast, the designed slide‐crosslinked polyrotaxane topological network (Figure 1c) achieves molecular‐level synergy between mechanical robustness and ion transport efficiency. As displayed in Figure 1c, the special dynamic structure of CD dissipates concentrated energy, while the static backbone of PMBA‐PPR_x_ maintains the overall stability of the electrolyte, achieving the collaborative optimization of mechanical and electrochemical properties at the molecular structure level.^[^ ^30^, ^31^ ^]^ Compared with the conventional crosslinked polymer electrolyte without the “pulley” molecule, the mechanically interlocked polymer electrolyte (PMBA‐PPR_x_) exhibits significantly enhanced dynamic adaptability. This improvement stems from CD‐based interlocking units within the crosslinked structure, where rotational and sliding motions of CD molecules effectively dissipate mechanical stress, which is verified by the following mechanical and electrochemical tests.

**Figure 2:**
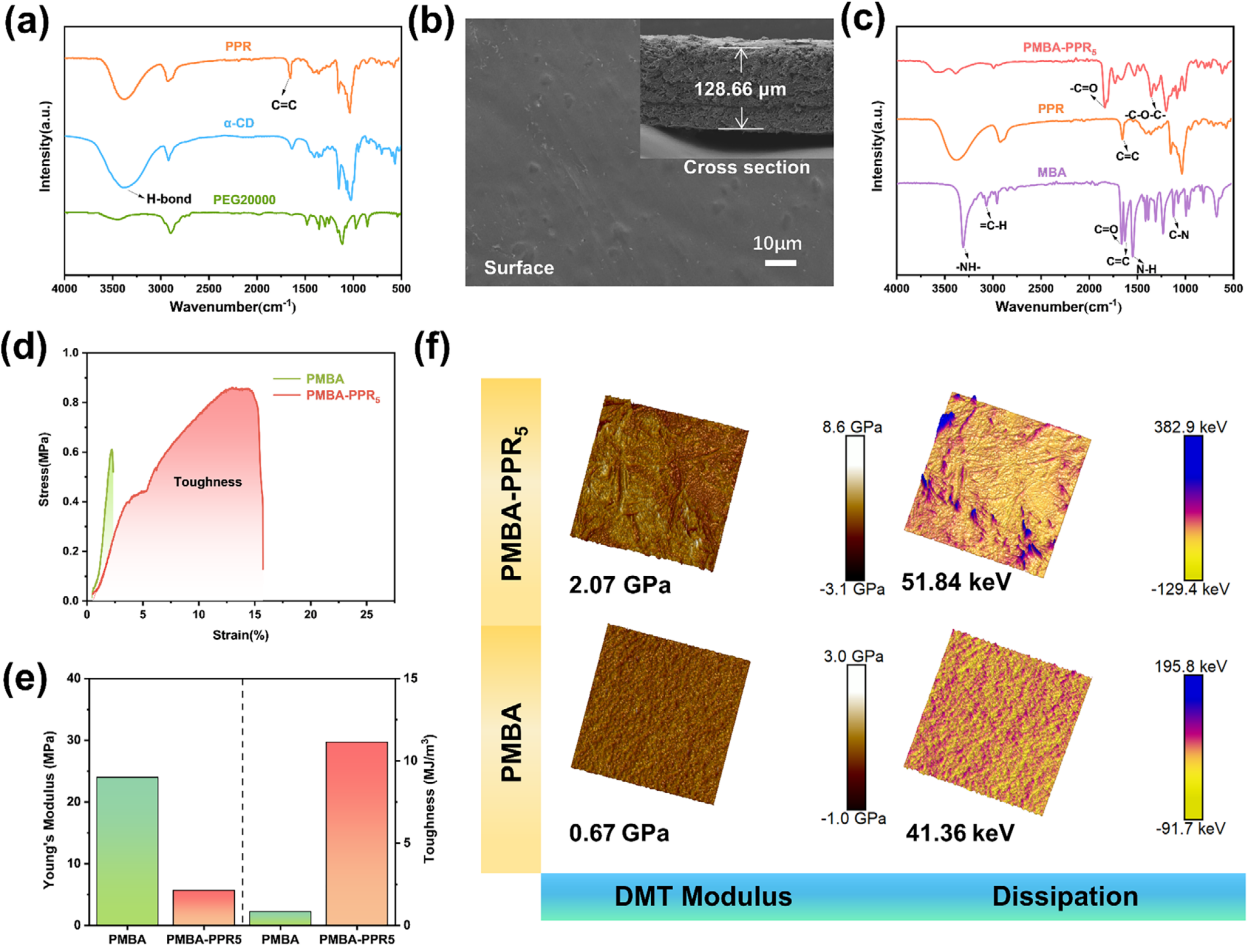
a) FTIR spectra of PPR, α‐CD, PEG20000. b) SEM images of PMBA‐PPR_5_. (The inset shows the cross‐section view of electrolyte.) c) FTIR spectra of PMBA‐PPR_5_, PPR, MBA. d) Stress–Strain curves of PMBA, PMBA‐PPR_5_. e) Comparison of mechanical performance of PMBA, PMBA‐PPR_5_. f) AFM modulus and dissipation diagrams of PMBA and PMBA‐PPR_5_.

As displayed in Figure S1 (Supporting Information), the synthesis of PMBA‐PPR_x_ electrolytes consists of two steps. The first step is the preparation of vinyl functionalized pseudorotaxane (PPRs), involving the self‐assembly behavior via host‐guest recognition of α‐CD and the modification of the ─OH groups of α‐CD and PEG (Figure 1a). The details are given in the supplementary materials. To confirm the successful preparation of the mechanically interlocked structure of PPRs, 2D nuclear Overhauser enhancement spectroscopy (NOESY) was applied along with the Nuclear Magnetic Resonance (NMR) analysis, as shown in Figure S2 (Supporting Information).^[^ ^32^ ^]^ As a powerful tool for the analysis of organic structure, NOESY spectra can reveal the spatial interaction between protons and can be used to judge the 3D structure of molecules.^[^ ^33^, ^34^ ^]^ The NOE signals with the blue rectangle(Figure S2a, Supporting Information) clearly showed the interaction between the PEG thread and the H_3,5,6_ of α‐CD, which confirms the formation of polymerizable pseudorotaxane (PPR). Obvious peaks in the chemical shift of a, b, c in the ^1^H NMR and an infrared stretching vibration peak at 1600 cm^−^¹ in Fourier transform infrared (FTIR) spectra(Figure S21b,c, Supporting Information; **Figure** **2**a) indicated that the ─OH groups of α‐CDs and PEG were successfully modified with vinyl groups.^[^ ^20^ ^]^

The second step is the synthesis of PMBA‐PPR_x_ (x = 1, 3, 5, 10, 20) electrolytes, as shown in Figure S3 (Supporting Information). The precursor solutions containing varying amounts of PPR initially existed as dilute solutions at room temperature. Through thermal radical polymerization at 80 °C for 6 h, these precursors became quasi solid electrolytes designated as PMBA‐PPR_x_. In the in‐situ polymerization, it is crucial to choose the suitable skeleton. As shown in Figure S4 (Supporting Information), PMBA‐PPR_5_ precursor exhibits a contact angle of 33.2° with glass fiber (GF) membranes, significantly lower than the contact angle (70.7°) with polypropylene (PP) separators. This substantial disparity in wettability indicates the better compatibility between the electrolyte precursor and the GF substrates. Therefore, GF membranes were selected as the skeleton for subsequent in‐situ polymerization processes.

The scanning electron microscopy (SEM) images in Figure 2b illustrated the surface and cross‐sectional micromorphology of the typical quasi‐solid electrolyte PMBA‐PPR_5_. As evidenced in Figure S5 (Supporting Information), the pristine GF membrane shows loose porous structure prior to the quasi‐electrolyte precursor incorporation. After the thermal polymerization, the resulting PMBA‐PPR_5_ displays a smooth and dense surface morphology with complete pore occlusion. It can be obtained from the cross‐sectional view that the thickness of the electrolyte is ≈128.66 µm. To verify the formation of crosslinked structure in PMBA‐PPR_5_, FTIR spectra were conducted (Figure 2c). Apparently, the absorption peaks at ≈1654 cm^−1^ correspond to the vibration of C═C groups in PPR. For the spectra of monomer MBA, the peaks at 1626 and 1540 cm^−1^ are assigned to the C═C and ═C─H, confirming the presence of reactive vinyl groups. After crosslinking, the vibrations of C═C and ═C─H disappeared, demonstrating that the PMBA‐PPR_5_ electrolyte was successfully synthesized.

Thermal stability serves as a crucial parameter for assessing the safety of electrolytes in practical applications. The thermogravimetric analysis (TGA) was used to measure the decomposition behavior of electrolytes. As shown in Figure S6 (Supporting Information), the thermal decomposition temperature of polymer framework exceeds 200 °C, exhibiting a two‐stage degradation process. The first stage (178–200 °C) corresponds to the decomposition of polymer framework itself, while the second stage (> 400 °C) arises from the decomposition of LiTFSI. Notably, the PMBA‐PPR_5_ exhibits enhanced thermal stability over the pristine PMBA, attributed to its stable crosslinked structure, and has the potential for use at higher temperature.^[^ ^35^ ^]^ The mechanical property of electrolytes is a crucial factor to restrain the lithium dendrites. Polymer electrolytes often struggle to withstand the stress generated by the dendrite growth s during the battery cycling process, thus breaking and causing internal short circuits in the battery. Through chemical crosslinking, the mechanical stability of polymer electrolytes can be significantly enhanced by establishing a crosslinked network structure. The tensile stress–strain curves of PMBA and PMBA‐PPR_5_ electrolytes are shown in Figure 2d. It can be seen that the addition of PPRs causes a certain increase in the mechanical properties of PMBA‐PPR_5_, which is ascribed to the mechanical interlocked structure of PPR. As shown in Figure 2d, PMBA‐PPR_5_ electrolyte provides a higher tensile strength (0.85 MPa) and elongation at break (15%) than PMBA electrolyte. Notably, the slide‐crosslinked structure in PMBA‐PPR_5_ reduces Young's modulus from 24 MPa for PMBA to 5.67 MPa while simultaneously achieving exceptional toughness (11.14 MJ m^−3^), which is enhanced by 1242% compared to PMBA (0.83 MJ m⁻^3^, Figure 2e). The results mainly ascribe to the intramolecular motions within rotaxane units upon external force thus toughening the slidable mechanically interlocked structure.^[^ ^36^ ^]^

Atomic force microscopy (AFM) was used to investigate the DMT modulus distribution ability of the PMBA‐PPR_5_ by using Peak Force quantitative nanomechanical (QNM) mode. Figure 2f exhibits the DMT modulus distribution images of PMBA‐PPR_5_ and PMBA, respectively. PMBA‐PPR_5_ exhibits much higher DMT modulus (2.07 GPa) than PMBA (0.67 GPa), with an indicator of superior ability of PMBA‐PPR_5_ to defend the growth of lithium dendrites.^[^ ^37^, ^38^, ^39^ ^]^ Additionally, the results demonstrate that PMBA‐PPR_5_ shows superior energy dissipation compared to PMBA, attributed to the dynamic mechanical interlocked structure that effectively alleviates the stress induced by the lithium dendrite growth. This enhanced energy dissipation capacity enables better accommodation of electrolyte volume variations during cycling.

### 2.2 Electrochemical Performance of PMBA‐PPR_x_ Electrolytes

**Figure 3:**
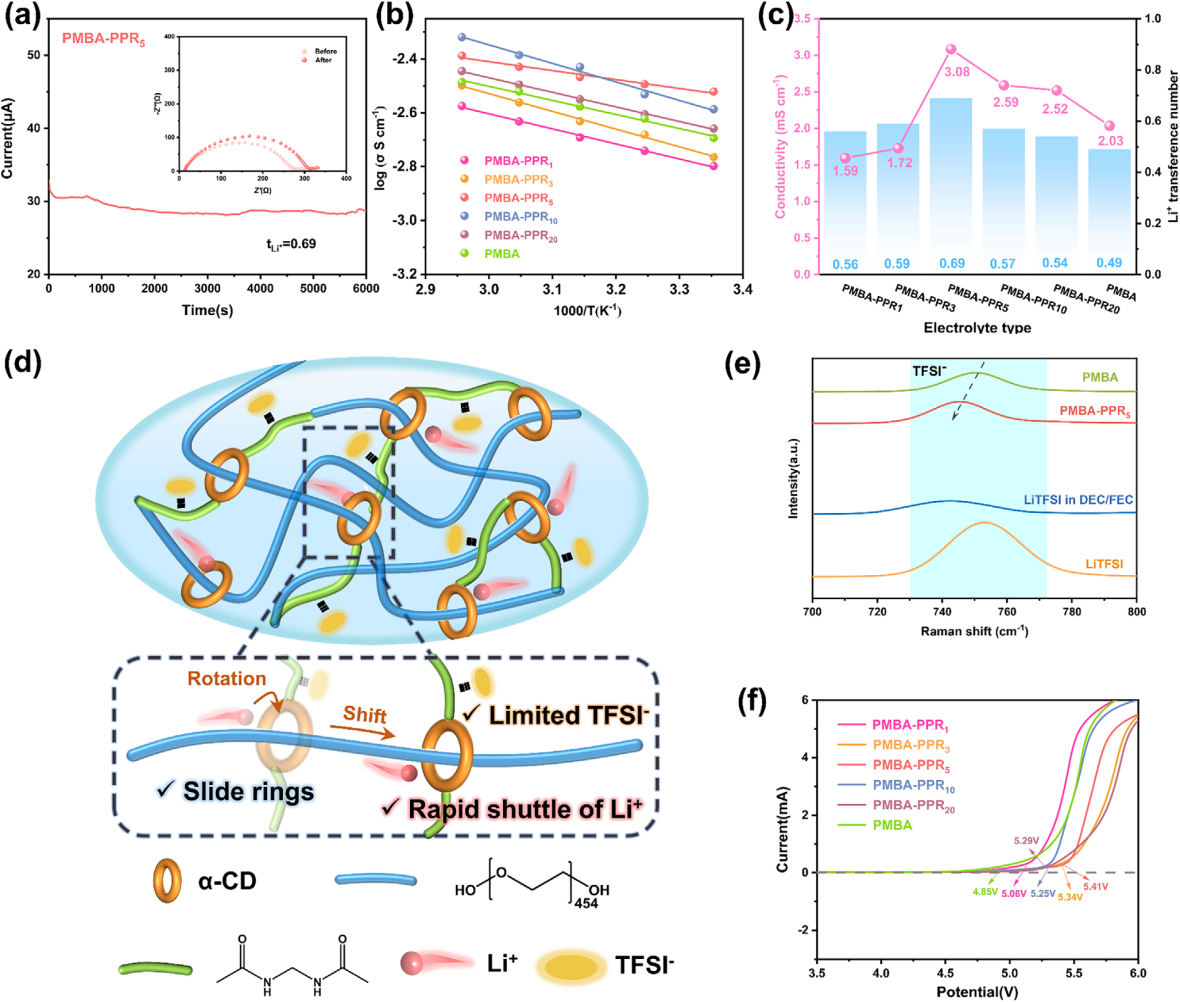
a) Chronoamperometry curves of the PMBA‐PPR_5_ electrolyte. (The insets show EIS before and after polarization.) b) Arrhenius spectrum corresponding to electrolyte. c) Relationship between ionic conductivity at room temperature and t_Li_^+^_._ d) Schematic of ion transport mechanism. e) Raman spectra of the electrolytes. f) LSV curves of electrolytes.

In order to explore the effect of PPR content on the electrochemical performance of electrolytes, we prepared a series of electrolytes with different PPR contents (Table S1, Supporting Information) named PMBA‐PPR_1_, PMBA‐PPR_3_, PMBA‐PPR_5_, PMBA‐PPR_10_, and PMBA‐PPR_20_, respectively. As shown in Figure S7 (Supporting Information). The precursor solutions with different PPR contents were in the state of dilute solutions at room temperature. With the increasing amount of PPR, the solution gradually changed from transparent to opaque, which indicated the reduction of the solubility of PPR. The Li^+^ transference number(t_Li_^+^) was obtained by chronoamperometry (CA) at 25 °C and the equivalent circuit for modelling EIS plots is shown in Figure S9 (Supporting Information).^[^ ^40^, ^41^, ^42^ ^]^ A higher t_Li_^+^ correlate with more Li^+^ participation in charge transference and mitigated concentration polarization during charging/discharging cycling. As shown in Figure S8 (Supporting Information) and **Figure** **3**a, PMBA‐PPR_5_ shows the highest t_Li_^+^ value (0.69) among the PMBA‐PPR_x_ electrolytes. In contrast, PMBA exhibits the lowest t_Li_^+^ value (0.49).

Using electrochemical impedance spectroscopy (EIS), the ionic conductivities of PMBA‐PPR_x_ electrolytes were determined at the temperature range of 25–65 °C (Figure S10, Supporting Information). The ionic conductivity of the electrolyte with temperature is fitted using the Arrhenius equation in Figure 3b, which suggests a synergistic ion conduction mechanism. According to the Arrhenius equation, the activation energy (E_a_) for Li^+^ migration in PBMBA‐PPR_5_ electrolyte is 0.15 eV, indicating a reduced energy barrier for Li^+^ migration upon PPR incorporation. As shown in Figure 3c, PMBA‐PPR_5_ electrolyte exhibits the highest ionic conductivity of near 3.08 mS cm^−1^ and Li^+^ transference number (0.69).

The integration of PPR into the PMBA crosslinked network increases the electrochemical performance of the electrolyte due to the dynamic sliding and rotating of α‐CD along the PEG chains. As illustrated in Figure 3d, this motion facilitates Li⁺‐ion dissociation via weakened α‐CD/Li⁺ coordination interactions, while the polar amide groups immobilize lager anions through strong electrostatic interactions, collectively promoting the Li^+^ transference efficiency. However, the excessive PPR content (> 5 wt.%) elevates crosslinking density, causing polymer chain entanglement, which is not conducive to the transport of lithium ions. Consequently, the optimal PMBA‐PPR_5_ electrolyte with 5% PPR demonstrates the best basic electrochemical properties, justifying its selection for subsequent comprehensive testing.

The dissociation behavior of Li⁺‐TFSI⁻ ion pairs in electrolytes were examined via Raman spectroscopy (Figure 3e). The stretching vibrations of N‐S bonds are observed at 754 cm^−1^ for TFSI^−^ in LiTFSI salt and 740 cm^−1^ for free state TFSI^−^ in DEC/FEC solutions, which are corresponded to associated and dissociated states. Apparently, the characteristic peaks belonging to PMBA and PMBA‐PPR_5_ are located at 751 and 744 cm^−1^, suggesting that the content of free‐state TFSI^−^ in PMBA‐PPR_5_ is much higher than that in PMBA. This result is attributed to the CD units in PPR that weaken Li^+^‐TFSI^−^ coordination through the complexation of hydroxyl groups with Li^+^, thereby facilitating the dissociation of lithium ions and regulating the transport of Li^+^. Significantly, the diameter of TFSI^−^ anions (7.9 Å) is much larger than the cavity size of α‐CDs (4.7–5.3 Å). Meanwhile, the polar amide groups on the electrolyte skeleton limit the movement of TFSI^−^. These two factors restrict the movement of TFSI^−^ anions while promoting selective Li^+^ transport.^[^ ^23^, ^43^ ^]^ According to Differential Scanning Calorimeter (DSC) results in Figure S11 (Supporting Information), PMBA‐PPR_5_ displays a lower glass transition temperature (T_g_, −39.8 °C), indicating better ability of segment relaxation. Moreover, X‐ray diffraction (XRD) curves in Figure S12 (Supporting Information) shows a wide diffraction peak of PMBA‐PPR_5_, a typical amorphous feature which is willing to the transference of Li^+^.

Linear sweep voltammetry (LSV) measurement was conducted to evaluate the electrochemical oxidation stability of electrolytes. As depicted in Figure 3f, the electrolyte oxidation voltage of PMBA‐PPR_1_, PMBA‐PPR_3_, PMBA‐PPR_5_, PMBA‐PPR_10_, PMBA‐PPR_20_ are 5.06, 5.25, 5.29, 5.34, and 5,41 V, respectively, which all exceed that of pristine PMBA (4.85 V). This improvement in oxidation stability is related to the integration of PPR within the crosslinked structure, which structurally suppress oxidative decomposition at high potentials. However, there is no linear relationship between the PPR contents and the increase of electrochemical window, and the electrochemical window of electrolytes with different PPR contents is concentrated in the range of 5.0–5.4 V, indicating that the PPR content has little effect on the oxidation stability of electrolytes.

To further investigate the oxidation stability of the electrolyte, electrochemical floating experiment was performed on Li||NCM811 cells containing PMBA and PMBA‐PPR_5_ electrolytes. As shown in Figure S13 (Supporting Information), the cells were charged to target voltages ranging from 4.2 to 5 V (vs Li^+^/Li), and kept at each potential for 2 h. Comparative analysis reveals that the PMBA electrolyte show a certain leakage currents exceeding baseline levels at voltages above 4.5 V, while the measured leakage current of PMBA‐PPR_5_ remained below 20 µA throughout 5 V, suggesting less interfacial side reactions in batteries.^[^ ^44^, ^45^ ^]^ These results provide compelling evidence that the designed dynamic crosslinking structure of PMBA‐PPR_5_ is effective in suppressing the oxidation reaction and affording high‐voltage battery operation.^[^ ^46^ ^]^

To investigate the role of PPR during the cycling process, Li|PMBA‐PPR_5_|LFP and Li|PMBA|LFP was assembled for electrochemical analysis. As illustrated in Figure S14a (Supporting Information), the cyclic voltammogram (CV) curves of PMBA‐PPR_5_ and PMBA were recorded within a voltage range of 2.5–4.3 V at a scan rate of 0.1 mV s^−1^. The potential gaps (∆V) between the oxidation and reduction peaks for PMBA‐PPR_5_ (0.30 V) is smaller than that of PMBA (0.45 V), indicating reduced polarization and enhanced reaction reversibility in the PMBA‐PPR_5_ system. Besides, the three CV curves of PMBA‐PPR_5_ are relatively overlapping in Figure S14b (Supporting Information), exhibiting well‐reversible electrochemical redox process.

**Figure 4:**
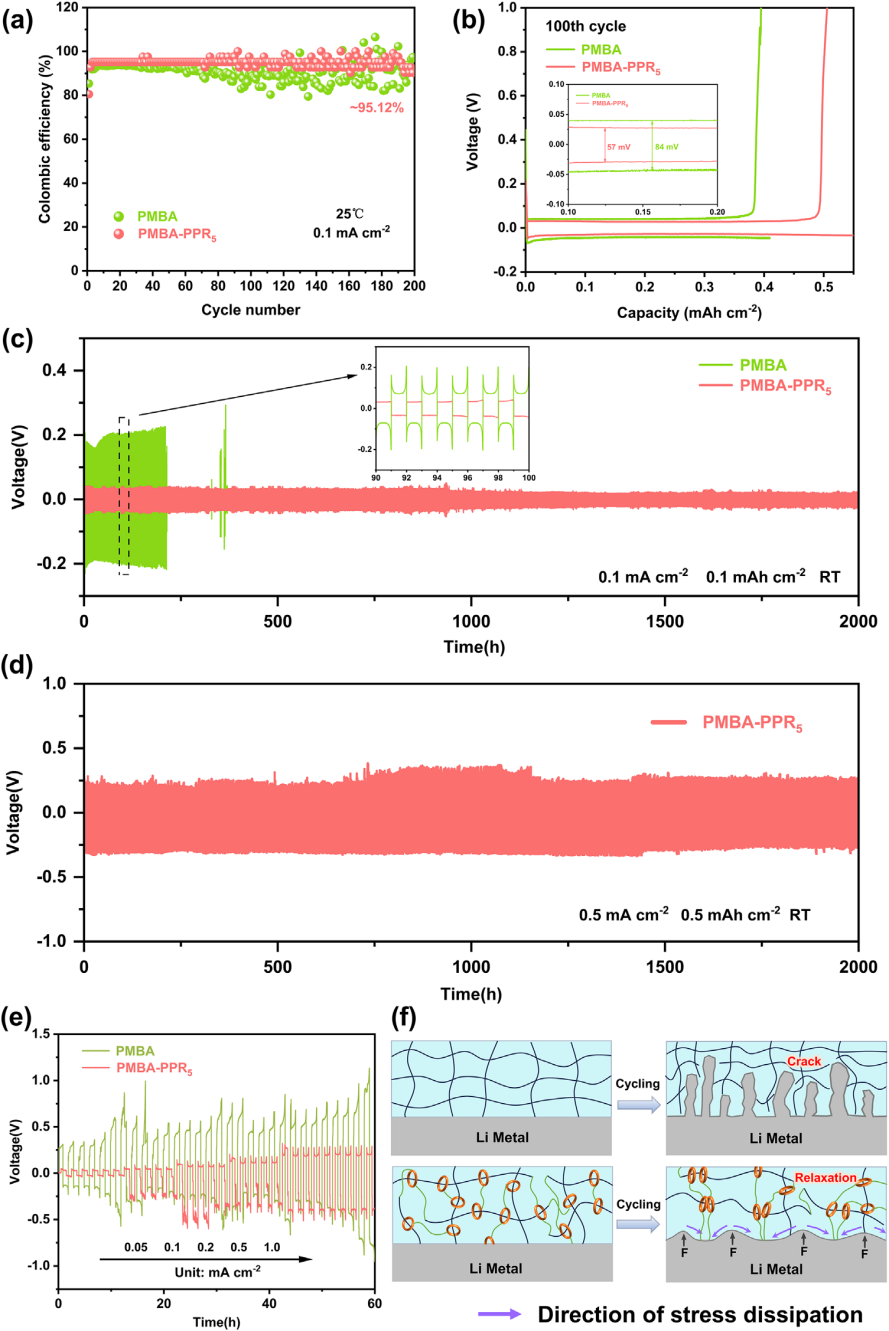
a) Coulomb efficiency of Li|PMBA|Cu and Li|PMBA‐PPR_5_|Cu half cells. b) Capacity‐voltage curves of the 100th cycle. c) Deposition/stripping curves of Li|PMBA|Li and Li|PMBA‐PPR_5_|Li cells at 0.1 mAh cm^−2^. d) Deposition/stripping curves of Li|PMBA‐PPR_5_|Li cell at 0.5 mAh cm^−2^. e) Voltage‐time curves of PMBA and PMBA‐PPR_5_. f) Schematic diagram of lithium deposition/stripping during cycling process.

To investigate the influence of electrolytes on lithium deposition/stripping behavior and interfacial stability, Li||Cu half cells were assembled for Coulomb efficiency (CE) test at a current density of 0.1 mA cm^−2^. As depicted in **Figure** **4**a, the PMBA‐PPR_5_‐based cell shows a stable cycle, maintaining a high and consistent CE of ∼ 95.12% over 200 cycles, indicating that adding PPR played a crucial role in building a stable solid‐electrolyte interphase (SEI). In contrast, the CE of the PMBA‐based cell deteriorates rapidly after 80 cycles, which is due to the fact that PMBA continues to react with the Li anode and decompose during the cycle, resulting in an unstable and nonuniform SEI layer. To further elucidate the structural advantage of PMBA‐PPR_5_ in regulating Li deposition, the voltage curves of PMBA‐PPR_5_ and PMBA are compared in Figure 4b. Remarkably, the PMBA‐PPR_5_ system retains a low deposition overpotential (∼ 57 mV) even after 100 cycles, indicating that the structure of PMBA‐PPR_5_ can build a rapid transmission channel of Li^+^ and a homogeneous Li^+^ flux distribution. This structural optimization significantly reduce local current density fluctuations, thereby inhibiting dendritic Li growth and enabling uniform Li^+^ deposition.^[^ ^47^ ^]^

To evaluate the long‐term interfacial stability and electrochemical performance, symmetric Li||Li cells with PMBA‐PPR_5_ and PMBA electrolytes were subjected to repeated deposition/stripping cycles. As shown in Figure 4c, the polarization voltage of the Li|PMBA‐PPR_5_|Li cell remains a minimal polarization voltage (∼ 0.02 V), over 2000 h of plating and stripping, while the polarization voltage of the Li|PMBA|Li cell displays significant voltage fluctuations after only 300 h, indicative of severe interfacial degradation. SEM is used to observe the surface of Li metal in Li||Li cells before and after cycling, as shown in Figure S15 (Supporting Information). It reveals that PMBA‐PPR_5_ effectively regulates the stripping and deposition of Li^+^, forming a smooth and stable interface. Remarkably, even at an elevated current density of 0.5 mA cm^−2^ (Figure 4d), the Li|PMBA‐PPR_5_|Li cell can still maintain stable plating/stripping for 2000 h with a consistently low polarization voltage (0.25 V). Although this value may seem relatively high, it is consistent with previous reports on quasi‐solid electrolytes, where similar polarization voltages ranging from 0.1 to 0.3 V have been observed under comparable conditions and even at higher testing temperature.^[^ ^48^, ^49^ ^]^ This slightly elevated polarization is attributed to the partially crosslinked polymer matrix, which moderately limits Li⁺ transport, as well as to possible interfacial activation processes during the initial cycles. Nevertheless, the stable voltage profile over prolonged cycling indicates sufficient electrochemical compatibility and interface stability of the electrolyte. The enhanced electrochemical stability of PMBA‐PPR_5_ was further validated through stepwise current density testing (Figure 4e), where the current density was incrementally increased from 0.05 to 1.0 mA cm^−2^. Owing to the outstanding interfacial compatibility of the PMBA‐PPR_5_ electrolyte, the symmetric Li||Li cell containing PMBA‐PPR_5_ electrolyte sustains a stable overpotential of ∼ 0.27 V even at 1.0 mA cm^−2^. Interestingly, the polarization voltage at 0.5 mA cm⁻^2^ in the stepwise rate test was lower than that at 0.5 mA cm⁻^2^ in the continuous current test. This is due to the limited duration of each current step in the rate test, which prevents the system from reaching steady‐state polarization. In contrast, the sustained current in constant‐current testing amplifies ion transport and interfacial limitations, resulting in higher polarization. Similar trends have been observed in previous reports on solid and quasi‐solid electrolytes.^[^ ^50^, ^51^, ^52^ ^]^ The PMBA‐based cell suffers from a gradually increasing overpotential during the plated/stripped process. These results verify the role of PPR‐based crosslinked polymer electrolyte in sustaining stable Li^+^ transport kinetics even at a higher current density (Figure 4f).

### 2.3 Performance of Full Batteries

**Figure 5:**
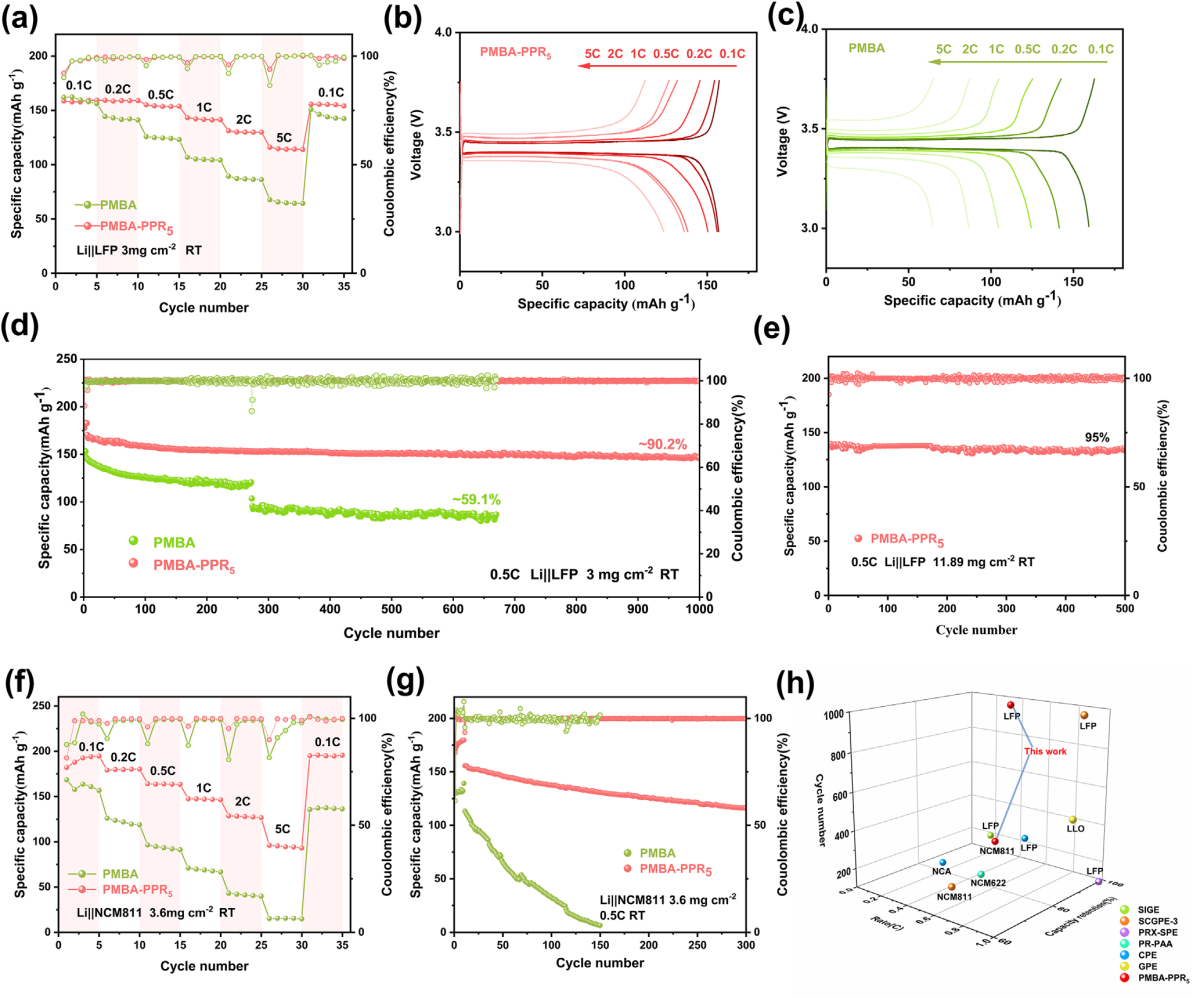
Li||LFP cells a) Rate performance of PMBA and PMBA‐PPR_5_ at 0.5 C; b) Capacity‐voltage curves of PMBA‐PPR_5_ at different cycles. c) Capacity‐voltage curves of PMBA at different cycles. d) Low active materials mass loading Li||LFP cells with PMBA‐PPR_5_ and PMBA cycle performance at 0.5 C. e) High active materials mass loading Li||LFP cells with PMBA‐PPR_5_ and PMBA cycle performance at 0.5 C. Li||NCM811 cells f) Cycle performance of PMBA and PMBA‐PPR_5_ at 0.5 C. g) Cycle performance of PMBA‐PPR_5_ and PMBA at 0.5 C. h) The comparison of the cycling performance of full cells assembled with PMBA‐PPR_5_ and other electrolytes reported in recent works(Table S2, Supporting Information).

**Figure** **5**a shows the rate performance of Li|PMBA‐PPR_5_|LFP and Li|PMBA|LFP. The Li|PMBA‐PPR_5_|LFP cell shows good charge‐discharge reversibility, demonstrating the structural and interfacial stability of the PMBA‐PPR_5_ electrolyte. Specifically, the Li|PMBA‐PPR_5_|LFP cell delivers discharge capacities of 159.3, 159, 153.7, 141.4, 129.8, and 113.9 mAh g^−1^ at 0.1 C, 0.2 C, 0.5 C, 1 C, 2 C, and 5 C, respectively, which are higher than those of the cells with PMBA electrolyte across all tested current densities. When the current rate is resorted to 0.1 C after high‐rate cycling, the Li|PMBA‐PPR_5_|LFP cell recovers to its original specific capacity without obvious degradation, confirming the fast Li^+^ transport pathways and the stable performance of PMBA‐PPR_5_ electrolyte. Figure 5b and shows the voltage curves of Li|PMBA‐PPR_5_|LFP and Li|PMBA|LFP at various current rates. It can be seen that the PMBA‐PPR_5_ electrolyte maintains a stable voltage platform without obvious polarization, while the PMBA electrolyte exhibits an obvious capacity reduction, indicating the electrochemical stability of the PMBA‐PPR_5_ during the rate charging and discharging process.

To evaluate the galvanostatic charge/discharge performance of Li||LFP full cells using PMBA‐PPR_5_ as the electrolyte, we assembled full cells with varying active material mass loadings and conducted electrochemical measurements at room temperature. Figure 5d presents the superior long‐term cycling stability of the PMBA‐PPR_5_‐based cell (active material mass loading, 3.0 mg cm^−2^) compared to PMBA electrolyte counterpart at 0.5 C. As the result, the PMBA‐PPR_5_ electrolyte enables stable operation over 1000 cycles and maintains a discharge specific capacity of 146.5 mAh g^−1^ with a capacity retention of 90.12%, accompanied by a consistently high coulombic efficiency more than 99.8%. In contrast, cells containing the PMBA electrolyte exhibit inferior cycling performance, displaying both lower initial specific discharge capacity and a significant decline in capacity after 250 cycles. This accelerated capacity fading is attributed to the interfacial side reactions, which is also reflected in the capacity‐voltage curves in Figure S16 (Supporting Information). Moreover, to validate the practical applicability of PMBA‐PPR_5_ electrolyte, we further investigated cells incorporating commercial‐grade cathode materials with high mass loading (11.89 mg cm⁻^2^). As shown in Figure 5e, the high‐loading cell maintains a discharge specific capacity of 139 mAh g^−1^ with 95% capacity retention for 500 cycles at 0.5 C under room temperature operation. Notably, Li |PMBA‐PPR_5_| LFP exhibited a stable specific discharge capacity at a high rate of 1 C. For temperature at 25 °C, the discharge specific capacity of the Li|PMBA‐PPR_5_|LFP cell after 100 cycles at 1 C is 123.7 mAh g^−1^, with a capacity retention rate of 94.7% (Figure S17, Supporting Information). This is due to the fact that the robust and flexible sliding crosslinked network increases the migration number of Li⁺ and the interfacial stability, thereby enhancing the ion transport and stress dissipation capabilities during the electrolyte cycling process, and demonstrating the potential of the electrolyte for application at higher rates. All these results collectively demonstrate the exceptional cyclic stability of PMBA‐PPR_5_ electrolyte.

Subsequently, the Li|PMBA‐PPR_5_|NCM811 full cell was assembled and tested to evaluate the high‐voltage cyclic performance of the PMBA‐PPR_5_ electrolyte. As shown in Figure 5f, the Li|PMBA‐PPR_5_|NCM811 cell exhibits remarkable rate capabilities with specific capacities of 188.2, 175.1, 160.9, 152.8, 125.1, and 100.2 mAh g^−1^ at 0.1 C, 0.2 C, 0.5 C, 1.0 C, 2.0 C, and 5.0 C rates, respectively, much higher than those of the Li|PMBA|NCM811. Furthermore, the Li|PMBA‐PPR_5_|NCM811 cell maintains a specific capacity of 116 mAh g^−1^ with a capacity retention rate of 74.5% after 300 cycles at 0.5 C (Figure 5g), as evidenced by the minimal polarization voltage evolution in the charge‐discharge curves (Figure S18, Supporting Information). Conversely, Li|PMBA|NCM811 cell shows obvious capacity attenuation during the cycle process, which cannot be well matched with high voltage cathodes for use. Figure 5h and Table S2 (Supporting Information) compare the cycling performance of full cell assembled with PMBA‐PPR_5_ and the full cells assembled with other different electrolytes reported in recently published works.^[^ ^23^, ^25^, ^30^, ^53^, ^54^, ^55^ ^]^

### 2.4 Electrochemical Stability and Interfacial Performances

**Figure 6:**
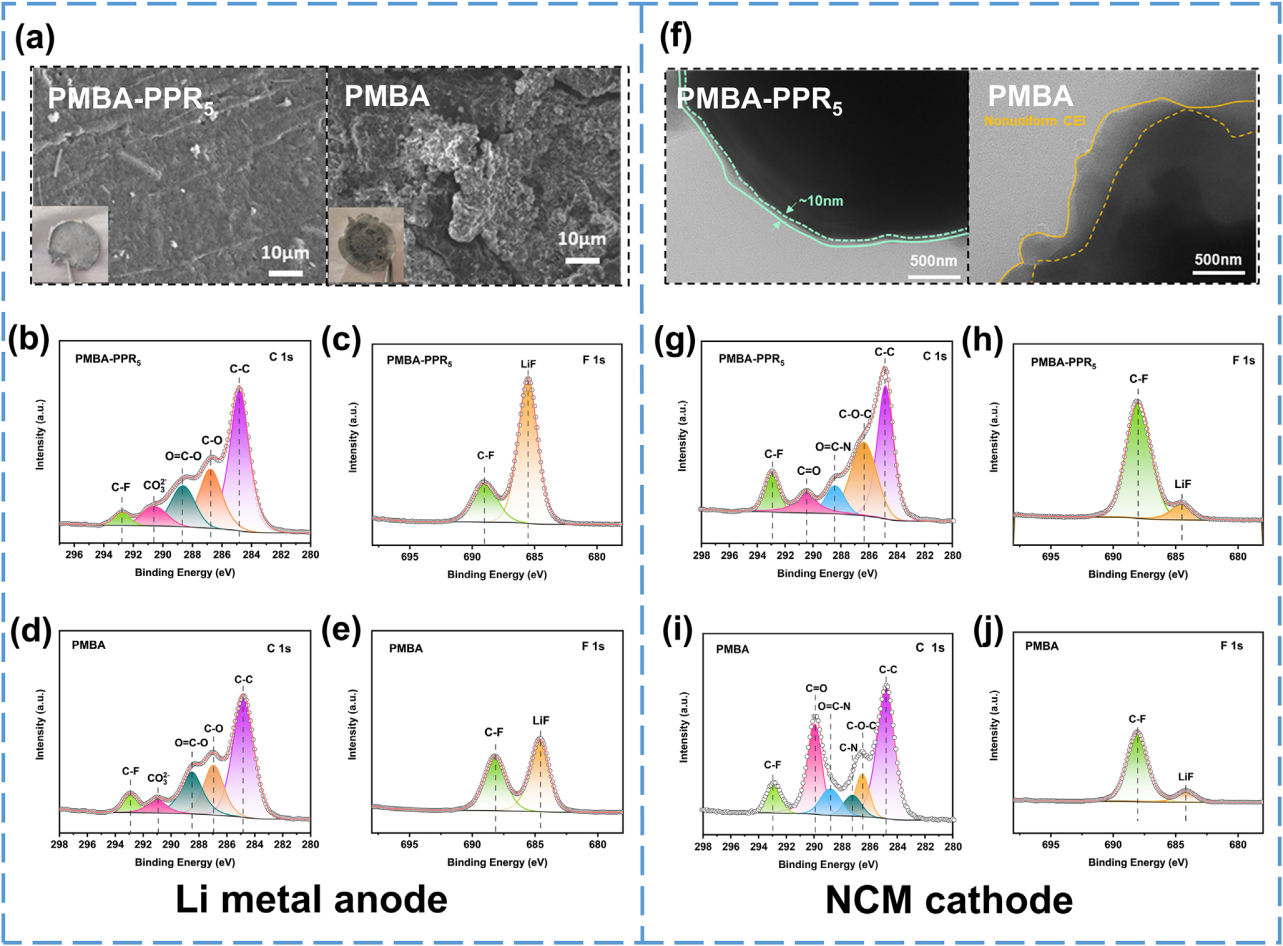
SEM images of Li anode surface from Li||NCM811 cells after 100 cycles. a) PMBA‐PPR_5._ (left) PMBA (right). XPS spectra of Lithium metal surface after 100 cycles: PMBA‐PPR_5_ b) C 1s. c) F 1s. PMBA(d) C 1s. e) F 1s. TEM images of NCM811 after 100 cycles. f) PMBA‐PPR_5._ (left) PMBA (right). XPS spectra of NCM811 after 100 cycles. PMBA‐PPR_5_g) C 1s. h) F 1s. PMBAi) C 1s. j) F 1s.

To further verify the excellent interface stability and lithium dendrite inhibition ability of the PMBA‐PPR_5_ electrolyte, the Li||NCM811 cell after 100 cycles at 0.5 C was disassembled for morphological characterization. SEM analysis shows a completely different lithium anode surface between PMBA‐PPR_5_ and PMBA electrolyte systems. As shown in **Figure** **6**a and S19 (Supporting Information), the lithium metal anode cycled with PMBA electrolyte shows a rough surface morphology featuring moss‐like protrusions, which is caused by uneven lithium dendrite deposition and interface side reactions. Relatively, the lithium metal anode assembled with the PMBA‐PPR_5_ electrolyte presents a relatively uniform and dense surface, demonstrating effective dendrite mitigation and stable SEI formation. These morphological observations visually confirm the role of PPR integration in enhancing the interfacial compatibility during battery cycle.^[^ ^56^ ^]^

To further verify the interfacial advantages of the PMBA‐PPR_5_ electrolyte, XPS analysis was performed on the cycled lithium metal anodes. Figure 6b–e and Figure S20 (Supporting Information) show the C 1s, F 1s, and N 1s spectra of lithium anode surfaces cycled with PMBA and PMBA‐PPR_5_ electrolytes. Obviously, five peaks located at C─C (284.8 eV), C─O (286.9 eV), O═C─O (288.2 eV), CO_3_^2−^ (291.1 eV) and C‐F (292.8 eV) are observed in Figure 6b,d. The PMBA‐PPR_5_ system exhibits reduced content of organic components in SEI layer, indicating that the crosslinked network can effectively inhibit the decomposition of polymer electrolyte. At the same time, stronger LiF (685.2 eV in F 1s) and Li_3_N (399.5 eV in N 1s) peaks are observed on the lithium metal surface of the PMBA‐PPR_5_ cell, indicating that the interface layer rich in inorganic substances are formed at the interface. This inorganic‐rich SEI reduces side reactions at the interface, improves interfacial mechanical properties, and promotes the internal stability of the cell.^[^ ^57^, ^58^ ^]^

The surface morphology and compositions of the cycled NCM811 cathode were also examined via TEM and XPS to analyze the changes in the CEI layer. As depicted in Figure 6f, TEM analysis of NCM811 particles after cycling reveals that the PMBA battery exhibits a thick and uneven CEI interface layer on NCM811 particles, whereas the PMBA‐PPR_5_ battery displays a thin and uniform CEI interface layer measuring ≈10 µm. Furthermore, XPS analysis demonstrates that the NCM811 cathode surface of the cell using PMBA‐PPR_5_ also possess an interface layer rich in inorganic substances, compared with the cell using PMBA (Figure 6g–j). The inorganic layer is mainly composed of LiF generated by TFSI^−^ anions decomposition.^[^ ^55^ ^]^ The presence of thin and uniform inorganic LiF layer not only mitigates side reactions at the electrode‐electrolyte interface, thereby preserving cathode structural stability, but also establishes a stable and rapid ion transport pathway, effectively enhancing battery cycle stability. In addition, the inorganic components are combined with a little number of organic components such as C─O─C (286.2 eV) and C═O (290 eV), which improves the toughness of the interface, enabling the interface layer to adapt to the volume change of the cathodes, and reducing the formation of cracks. On the contrary, the CEI interface layer in PMBA cell contains more organic components, as evidenced by heightened C─O/C═O peak intensities (Figure S21, Supporting Information). This organic‐rich interface exhibits poor thermal and chemical stability, demonstrating a propensity for oxidative decomposition. Such inherent instability renders the interface ineffective in safeguarding the cathode materials, thereby accelerating the structural degradation of the positive electrode, and thus leading to the decline of battery capacity. In general, in the Li|PMBA‐PPR_5_|NCM811 cells, the combination of a large amount of LiF inorganic components and a small amount of organic components can form a stable and high‐voltage resistant interface, which effectively optimizes the cycle and rate performance of the battery.^[^ ^59^, ^60^ ^]^

## 3 Conclusion

In this work, we rationally designed a slide‐crosslinked polymeric quasi‐solid electrolyte (PMBA‐PPR_5_) through in situ polymerization of reasonable content of vinyl functional polyrotaxanes (PPRs) and N,N'‐methylenebisacrylamide (MBA), achieving an optimized balance between mechanical property and ionic transport. The optimized PMBA‐PPR_5_ electrolyte exhibits a stable topological network and demonstrates exceptional electrochemical performance, including a wide electrochemical stability window (up to ∼ 5.5 V), a high Li^+^ transference number (0.67), and enhanced ionic conductivity (3.28 mS cm^−1^). The slide‐crosslinked structure of the PMBA‐PPR_5_ electrolyte endows its toughness to be 1242% higher than that of the PMBA electrolyte, enabling stable interfacial compatibility. Therefore, the PMBA‐PPR_5_ electrolyte reveals outstanding cycling stability when applied for high mass loading LFP cathode (11.89 mg cm^−2^) retaining the capacity retention of ∼ 95% after 500 cycles at 0.5 C. Moreover, the assembled Li|PMBA‐PPR_5_|NCM811 cell shows stable cycling performance with the capacity retention of ∼ 74.5% after 300 cycles at 0.5 C. This work provides an effective principle for in situ polymerized electrolytes to address critical challenges in high‐voltage lithium metal batteries through synergistic topological engineering of mechanical robustness and electrochemical stability.

## 4 Experimental Section

All experimental details are shown in the Supporting Information.

## Conflict of Interest

The authors declare no conflict of interest.

## Supporting information



Supporting Information

## Data Availability

The data that support the findings of this study are available from the corresponding author upon reasonable request.
