# Supplementary material for: Slide‐Crosslinked Polyrotaxane Topological Networks: Quasi‐Solid Electrolyte for High‐Voltage Lithium Metal Batteries
Source: Adv Sci (Weinh). 2025 Jul 17;12(39):e08598. doi: 10.1002/advs.202508598 (PMC12533377; doi:10.1002/advs.202508598)
Supplement: Supplementary file 1 — Supporting Information [file ADVS-12-e08598-s001.docx]

**Supporting Information**

**Slide-crosslinked** **Polyrotaxane Topological Networks: Quasi-solid Electrolyte for High-voltage Lithium Metal Batteries**

*Huirong Zhu^a^, Xiaoyue Zeng^a^, Xuewei Liu^a^, Jiaxing Zhu^a^, Jinghao Hua^a^, Jinle Lan^a^ *, Yunhua Yu^a^ *, and Xiaoping Yang^a^*

^a^ State Key Laboratory of Organic-Inorganic Composites, College of Materials Science and Engineering, Beijing University of Chemical Technology, North Third Ring Road 15, Chaoyang District, Beijing 100029, P. R. China

**Corresponding author:* [*lanjl@mail.buct.edu.cn; yuyh@mail.buct.edu.cn*](mailto:lanjl@mail.buct.edu.cn;%20yuyh@mail.buct.edu.cn)

**Materials**

α-Cyclodextrin (α-CD), Polyethylene glycol (PEG, MW=20000), 2-Isocyanatoethyl Acrylate (AOI), Dibutyltin dilaurate (DBTDL), Butylated Hydroxytoluene (BHT), 2,2-Azobisisobutyronitrile (AIBN), N-methyl-2-pyrrolidone (NMP), Dimethyl sulfoxide (DMSO) and N,N'-methylene diacylamine (MBA) were purchased from Aladin Co. Ltd. Glass Fiber (GF/A) membrane was purchased from Whatman. Bis(trifluoroethane) sulfonamide lithium (LiTFSI), Diethyl carbonate and Fluorinated Ethylene Carbonate (DEC:FEC=2:1, v/v%) were purchased from DoDo Chem. Polyvinylidene fluoride (PVDF), lithium iron phosphate (LiFePO_4_), and Super P, and LiNi_0.83_Mn_0.07_Co_0.1_O_2_ and commercial-grade cathode materials (mass loading 11.89 mg cm^-2^) were purchased from Canrd. All the above chemicals were used directly as purchased without further purification treatment.

**Materials characterization**

^1^H NMR spectra and 2D NOESY ^1^H NMR spectra were recorded on a BRUKER ADVANCE Ⅲ 600MHz spectrometer. Fourier-transform infrared (FTIR) spectra were recorded on a Nicolet 8700 FT-IR spectrometer. The contact angel was measured by a contact angel measuring equipment. The thermal stability of electrolytes was conducted using a Thermogravimetric Analyzer (TGA) in the temperature range from 25 ℃ to 600 ℃ under the N_2_ atmosphere at a heating rate of 10℃ min^-1^. Differential Scanning Calorimeter (DSC) analysis was carried out in the temperature range from -60℃ to 60℃ at a heating rate of 10 ℃ cm^-1^. X-ray diffraction (XRD) was characterized on a Bruker D8 by Cu Kα radiation. X-ray photoelectron spectroscopy (XPS) was employed to detect the surface chemical compositions by using Al Kα radiation. The surface morphologies of electrolytes and Li anodes were observed by scanning electron microscopy (JEOL JSM-7800F). The stress-stain curves of electrolyte specimens (50 mm×10 mm) were obtained by the tensile test on a universal testing machine at a crosshead speed of 4mm min^-1^. The thickness of the specimen was measured using a thickness gauge. Atomic Force Microscopy (AFM) was performed on a Bruker Dimension Icon to examine the Young's Modulus of the electrolytes. Raman spectra were carried out by a Horiba LabRAM HR Evolution from 100~4000 cm^-1^ with a 785 nm excitation wavelength.

**Electrochemical characterization**

Constant current cycling and multiplication performance of solid polymer electrolyte-based solid-state Li metal batteries were measured using a battery test system (LAND CT2001A). The cycling stability of PMBA and PMBA-PPR_x_ electrolytes against Li metal was measured with Li||Li symmetric cells at a fixed capacity density of 0.1 mA cm^-2^ and 0.5 mA cm^-2^ for 1 h, respectively. Electrochemical tests of the Li||LFP and Li ||NCM811 batteries were performed at room temperature in the 2.75-3.75 V voltage range and 3.0-4.3 V, respectively.

The ionic conductivity of the SPEs was evaluated using electrochemical impedance spectroscopy (EIS) on Auto Lab electrochemical workstation in the range of 25 °C to 65 °C, at frequencies from 1 MHz to 0.1 Hz, with an alternating current amplitude of 5 mV. The ionic conductivity was measured by assembling SS||SS cells, which were kept warm for 30 min before the start of each test temperature to reach thermodynamic equilibrium. The ionic conductivity (𝜎) was calculated according to the following Equation (S1):

$\text{σ=}\frac{\text{L}}{\text{A×R}}$ ...............................................Equation (S1)

where, *R* is obtained from AC impedance spectroscopy, *L* and *A* are the thickness and area of the SPEs.

The activation energy for Li^+^ transport through SEI was based on the temperature-dependent EIS measurement of SS|| SS cells (25 ~ 65 °C). The activation energy can be obtained according to the slope of the linear fit of the Arrhenius plot. The Arrhenius equation is shown as the following Equation (S2):

$\text{E}_{\text{a}}\text{=RTln}\frac{\text{A}}{\text{σ}}$......................................... Equation (S2)

Where, 𝜎 is the ionic conductivity, *A* is the pre-reference factor, *R* is the Universal gas constant, and *T* is the absolute temperature.

The Li-ion transference number (t_Li_^+^) was tested in a symmetric lithium cell using an Auto Lab at room temperature by combining DC polarization and AC impedance technique. A small DC potential (*∆V*=10 mV) was applied for 3000 s to gain the initial and steady currents. Meanwhile, the AC impedance spectra of the same cell were measured before and after polarization. The Li-ion transference number was calculated by the Bruce-Vincent-Evans Equation as shown in the following Equation (S3):

$t_{{Li}^{+}}=\frac{I_{s}\left( \Delta V-I_{0}R_{0} \right)}{I_{0}\left( \Delta V-I_{s}R_{s} \right)}$ ..................................Equation (S3)

Where, *I_0_* and *I_s_* are the initial and steady-state DC currents, *R_0_* and *R_s_* are initial and steady-state interface resistances, respectively. *∆V* is the polarization potential set at 10 mV.

The electrochemical window of the electrolyte was measured using linear scanning voltammetry (LSV), where a stainless steel was used as the working electrode and Li foil was used as the reference electrode. The voltage was increased from open circuit to 6 V using the Auto Lab Electrochemical Workstation with a scanning rate of 5 mV s ^-1^. Cyclic voltammetry (CV) was conducted on Auto Lab Electrochemical Workstation over a voltage range of 2.75-3.75 V for Li||LFP full cells, 3.0-4.3 V for Li||NCM811 full cells and with a scan rate of 0.1 mV s^-1^.

The Coulombic efficiency of Li metal anodes was accurately determined using a modified Auerbach method, implanted in Li||Cu cells through lithium deposition/stripping cycling. In each cycle, 0.1mAh cm^-2^ of lithium was deposited on the Cu electrode at a current density of 0.1 mA cm^-2^, followed by a stripping process terminated when the cell potential reached 1.0 V vs. Li/Li^+^.

**Synthesis of PMBA and PMBA-PPR electrolytes**

**Preparation of** **vinyl functionalized pseudorotaxane (PPRs)**: First, PEG20k was fully dried at 50 ℃ under vacuum for 12 h. Then, PEG20k and α-CD (α-CD : PEG20k = 8 : 1, molar ratio) were dissolved in 30 ml of distilled water and stirred at 400 rpm and 80 ℃ for 2 h. The solution was cooled down to room temperature and kept at 5℃ overnight to obtain the white paste. The paste was fully dried in a freeze dryer for 48 h to obtain the white powder, named pseudorotaxane. Then, 13.5g pseudorotaxane was dissolved in DMSO at 80 ℃ for 30 min. After that, 9 drops DBTDL and 1 wt.% BHT (relative to AOI) was added and the resulting mixture was further stirred at 50 ℃ for 30 min. Subsequently, 6.424 g AOI was dissolved in DMSO, which was then gradually added to the previous mixed solution and reacted continuously for 48h at 40 ℃. Finally, the resulting product was precipitated from acetone and was washed repeatedly with acetone three times. The collected paste was fully dried in a freeze dryer for 48 h to obtain the white powder, named vinyl functionalized pseudorotaxane (PPRs). All the steps were reacted at N_2_ atmosphere.

**Preparation of PMBA and PMBA-PPR_x_ electrolyte**: The quasi-solid electrolyte was obtained via in situ thermal polymerization of the precursor mixture solution. First, certain mass ratio (Table S1) of MBA, LiTFSI, PPR, and AIBN initiators were dissolved into the mixed solvent of DEC and FEC (2 : 1 wt%) and subsequently stirred to obtain a homogeneous precursor solution. Next, the precursor solution was injected into a glass fiber membrane in a coin cell. The assembled cells were subsequently heat at 80 ℃ for 6 h to guarantee full polymerization. All procedures were executed in Ar-filled glove box with the oxygen and moisture contents below 0.5 ppm.

**Preparation of Cathodes:** The LFP and NCM811 cathodes were prepared via the slurry casting process with a mass ratio of active materials: Super P : PVDF binder = 8 : 1 : 1. The homogenized slurry were coated onto carbon-coated aluminum foil and then dried at 80 °C for 24 h in a vacuum. Subsequently, the cathode was cut into discs with a diameter of 12 mm by punching machine, and the active materials mass loading is ~ 3.0 mg cm^−2^ for the LFP and ~ 3.6 mg cm^−2^ NCM811 cathodes.

**Table S1** Electrolyte ratio table with different PPR content

| Electrolyte | PPR(wt% relative to DEC/FEC) | MBA(wt% relative to DEC/FEC) | AIBN(wt% relative to monomer) |
| --- | --- | --- | --- |
| PMBA-PPR_1_ | 1 | 10 | 0.5 |
| PMBA-PPR_3_ | 3 | 10 | 0.5 |
| PMBA-PPR_5_ | 5 | 10 | 0.5 |
| PMBA-PPR_10_ | 10 | 10 | 0.5 |
| PMBA-PPR_20_ | 20 | 10 | 0.5 |
| PMBA | 0 | 10 | 0.5 |

**Supplementary Figures**


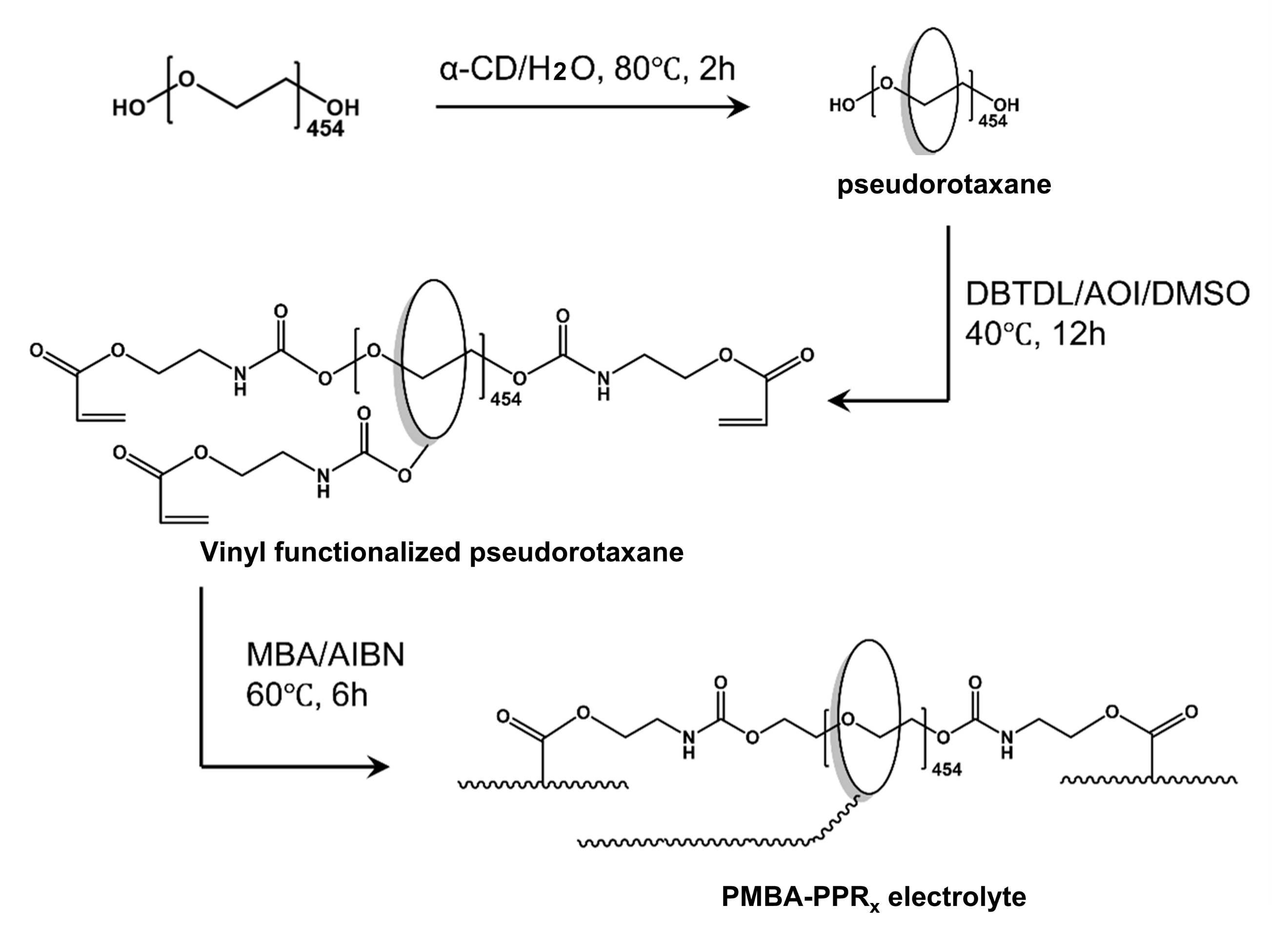


**Figure S1** The preparation process diagram of vinyl functionalized pseudorotaxane (PPRs) and PMBA-PPR_x_ electrolyte.


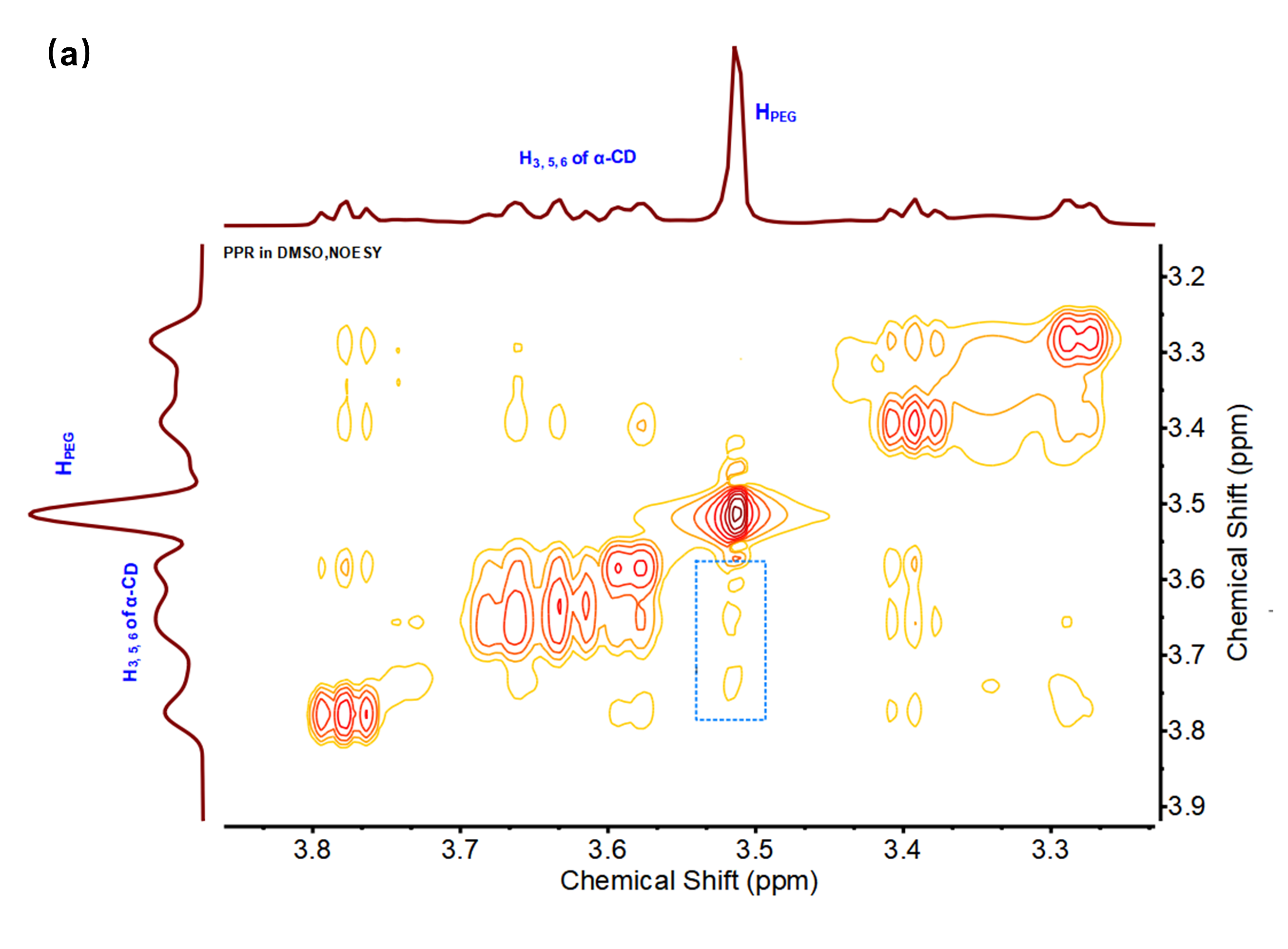


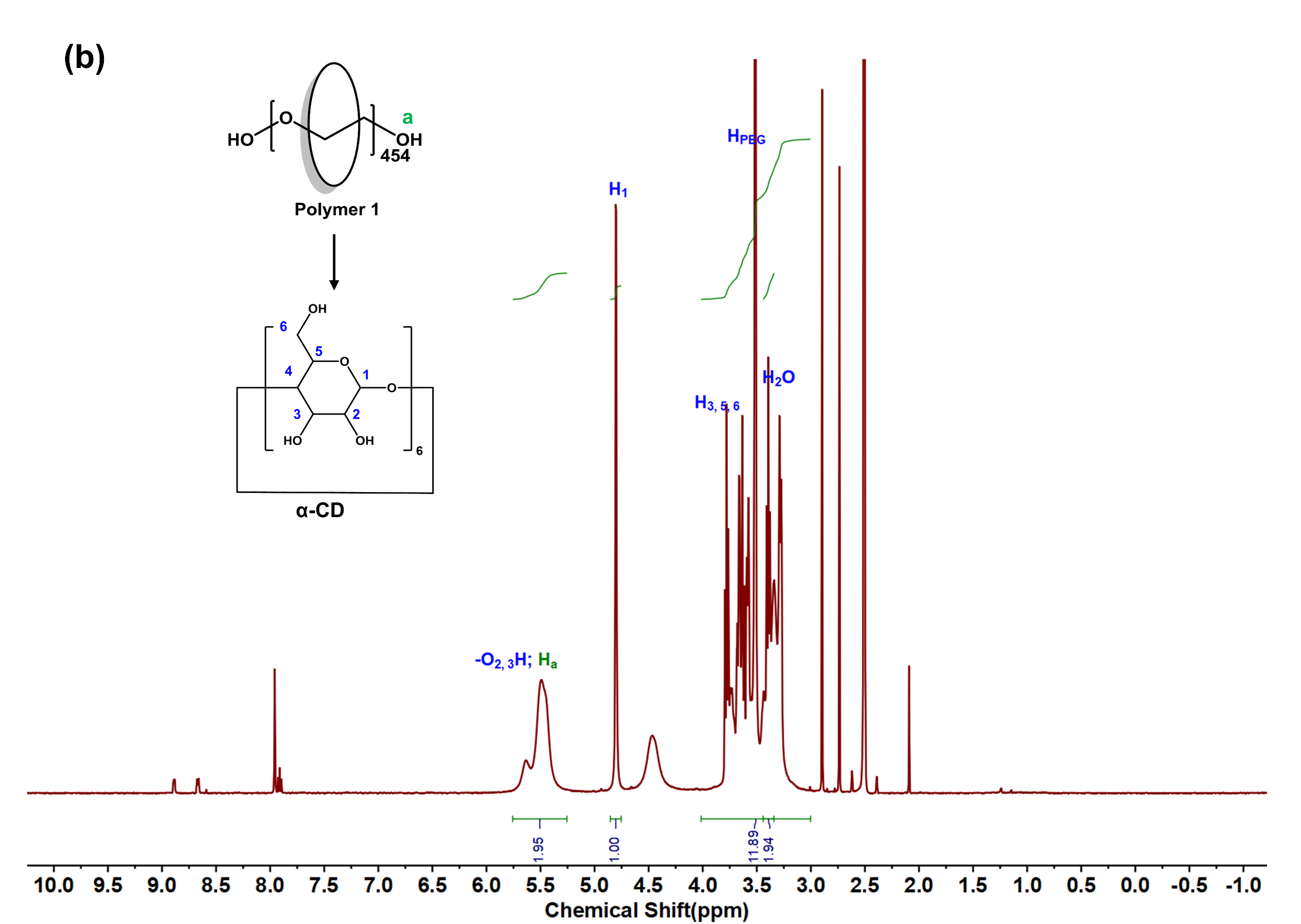


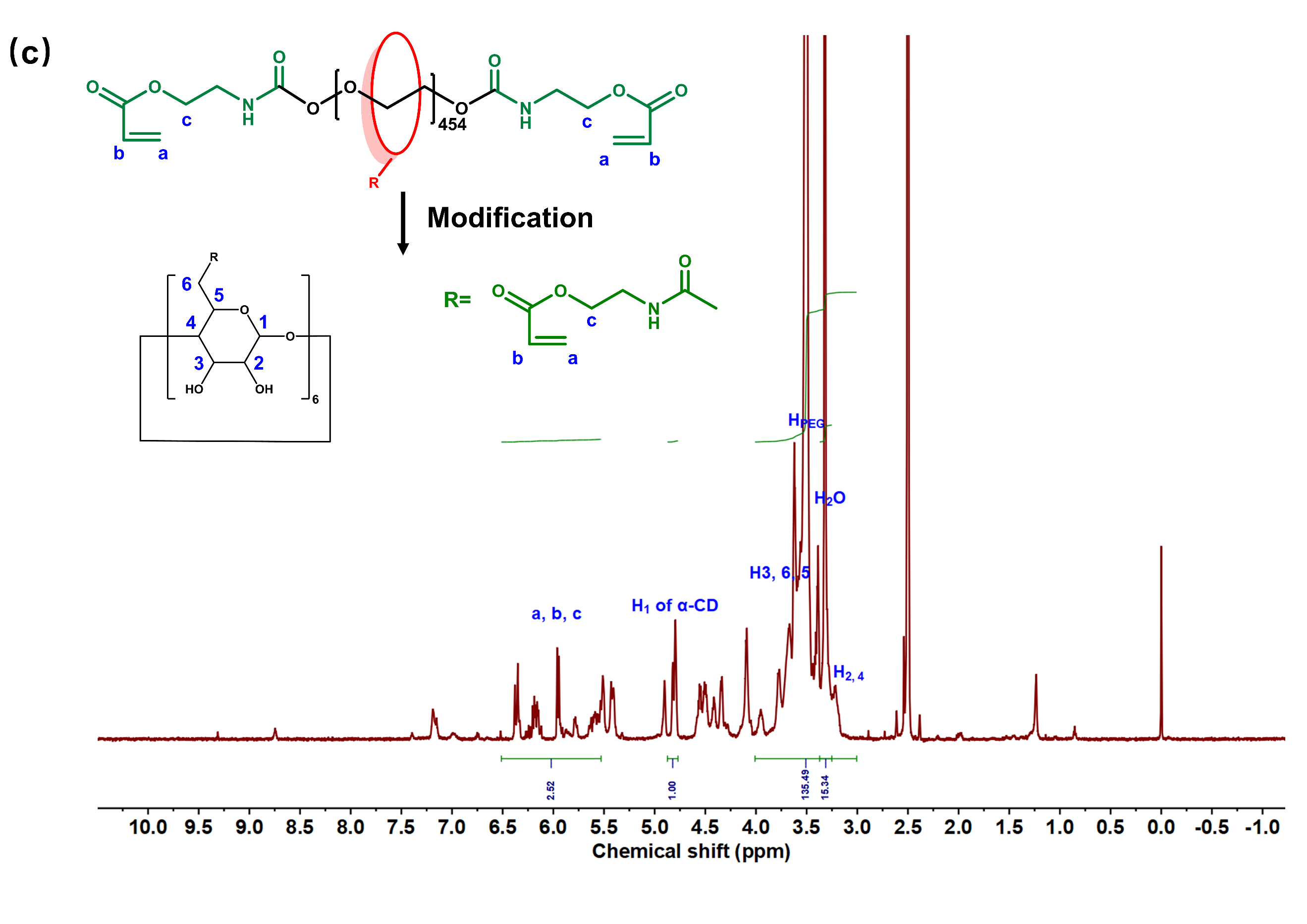


**Figure S2** (a) 2D NOESY spectra of PPR. (b) ^1^H NMR of pseudorotaxane. (c) ^1^H NMR of PPR.


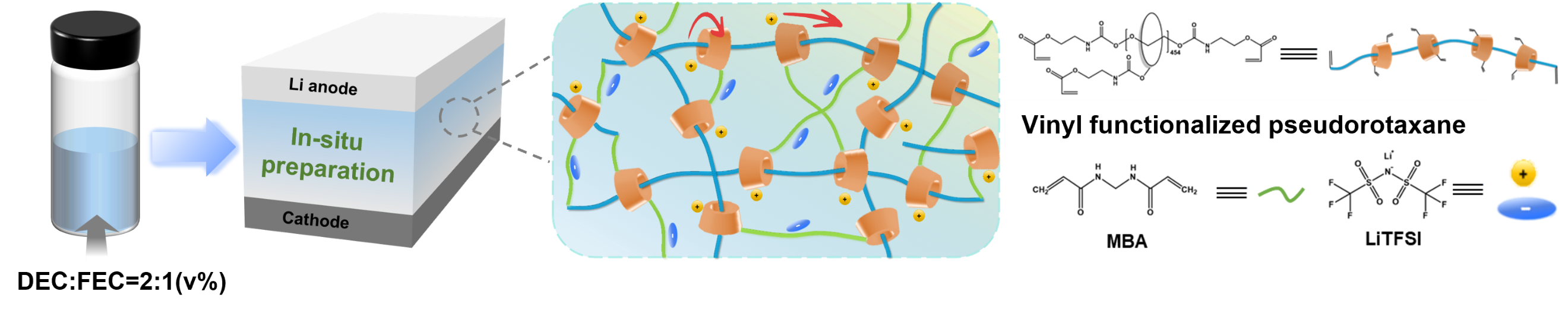


**Figure S3** In situ preparation process diagram of PMBA-PPR_x_ electrolytes.


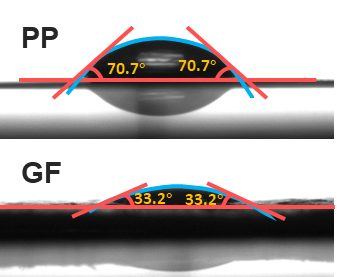


**Figure S4** Contact Angle test of PMBA-PPR_5_ precursor solution.


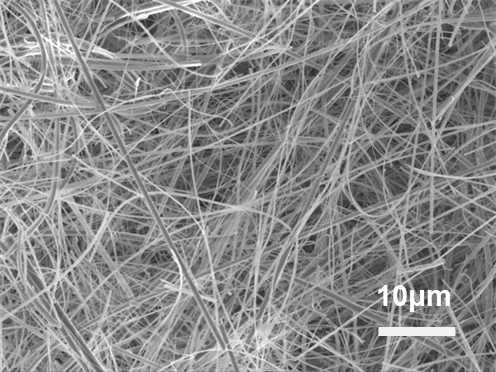


**Figure S5** SEM image of GF membrane.


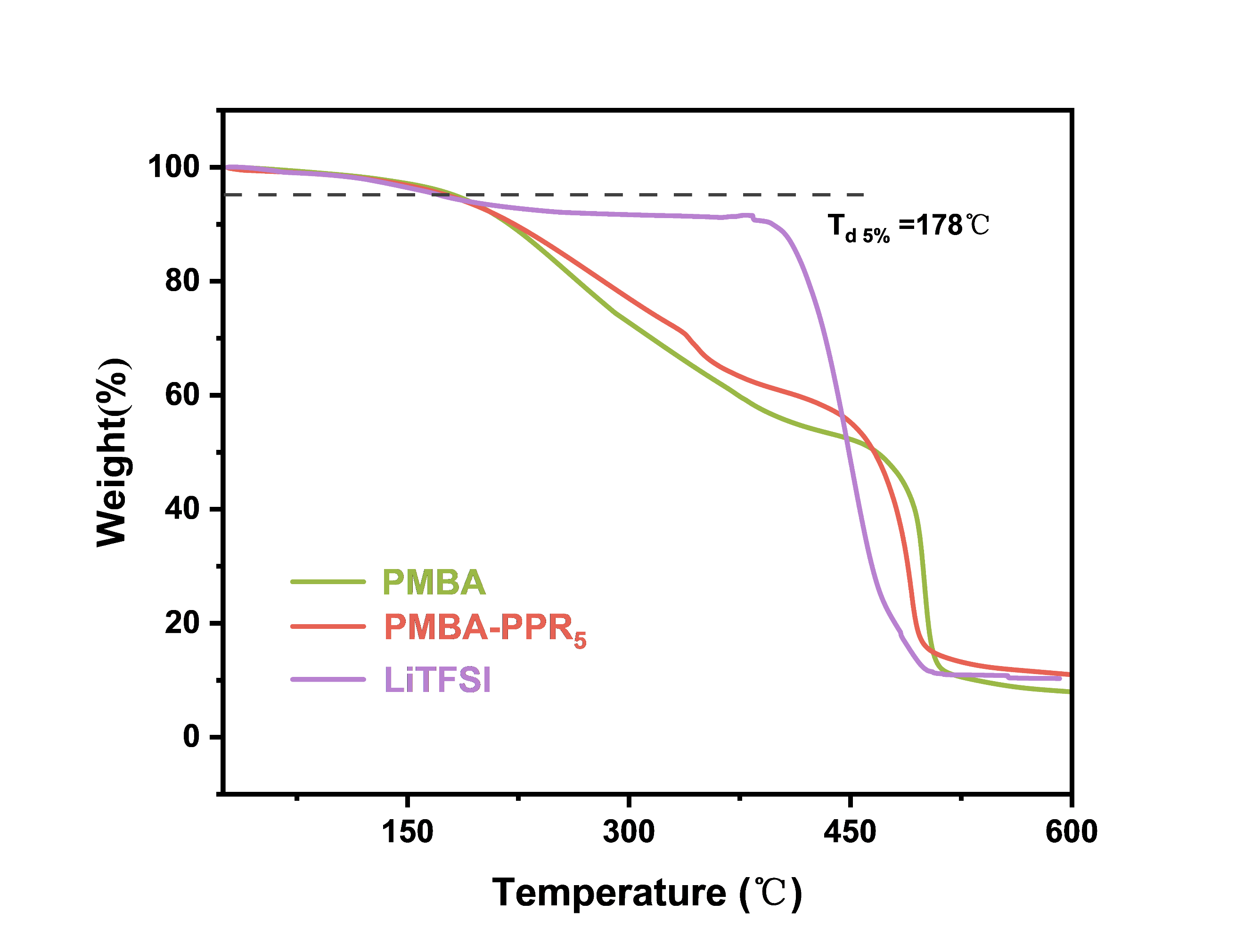


**Figure S6** TGA curves of LiTFSI, PMBA, and PMBA-PPR_5_ after in situ polymerization. The thermal decomposition temperatures of SPEs were higher than 200 ℃, which greatly guaranteed the safety of LMBs in practice use.


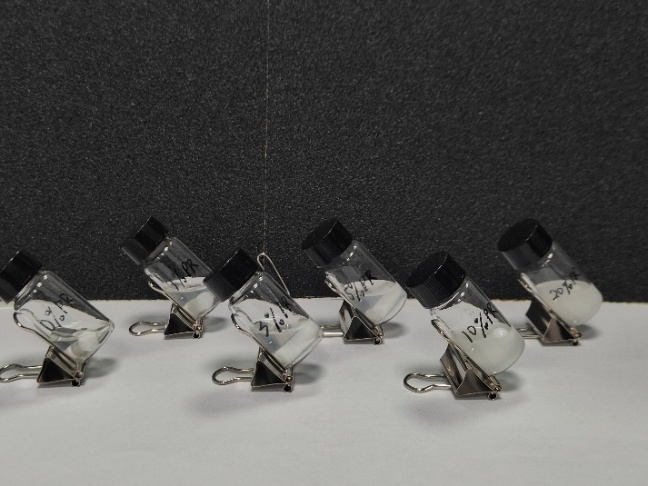


**Figure S7** Photos of electrolyte precursor solutions with different PPR contents.


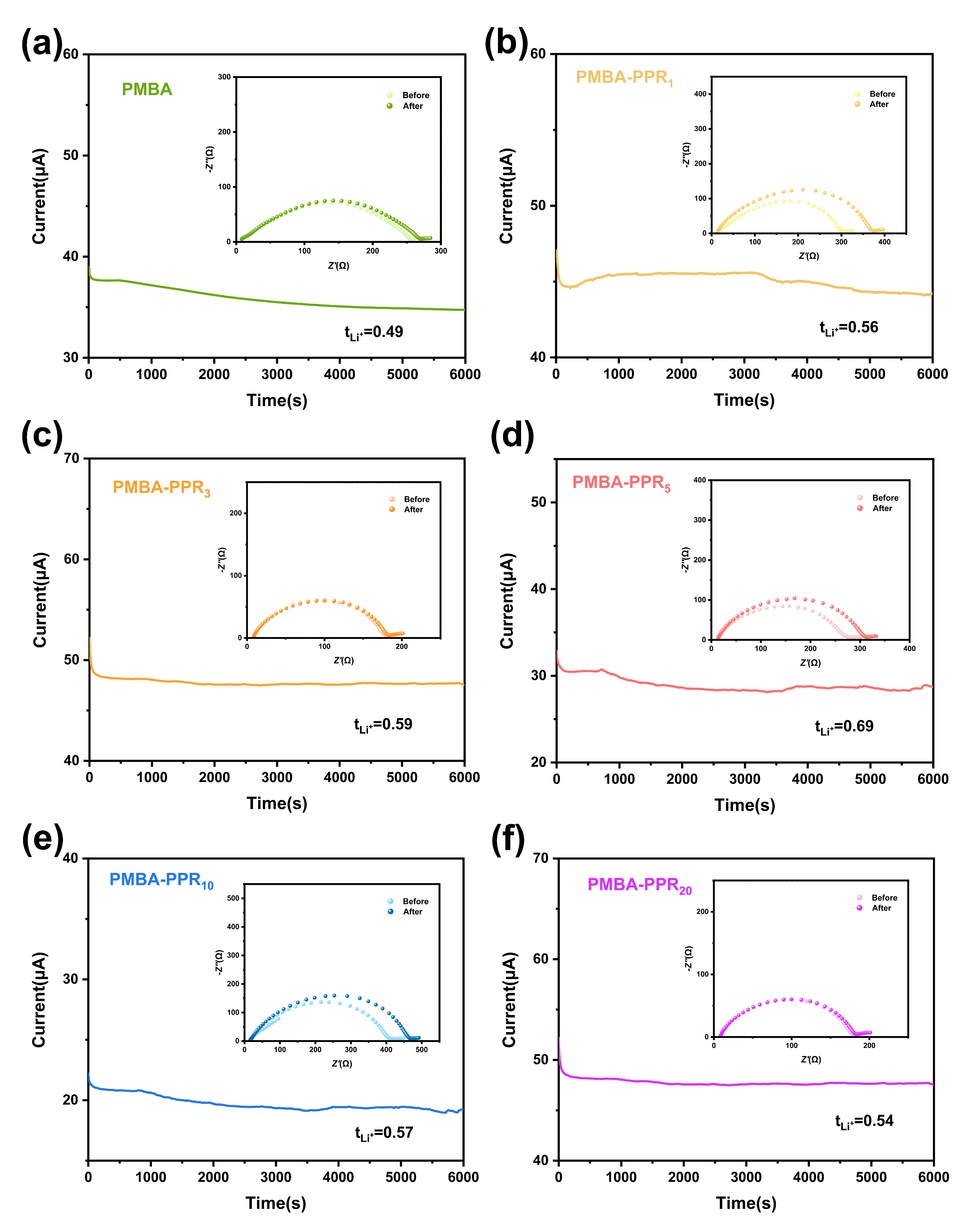


**Figure S8** Chronoamperometry curves of the electrolytes. (a)PMBA. (b)PMBA-PPR_1_. (c)PMBA-PPR_3_. (d)PMBA-PPR_5_. (e)PMBA-PPR_10_. (f)PMBA-PPR_20_. (The insets show EIS before and after polarization).

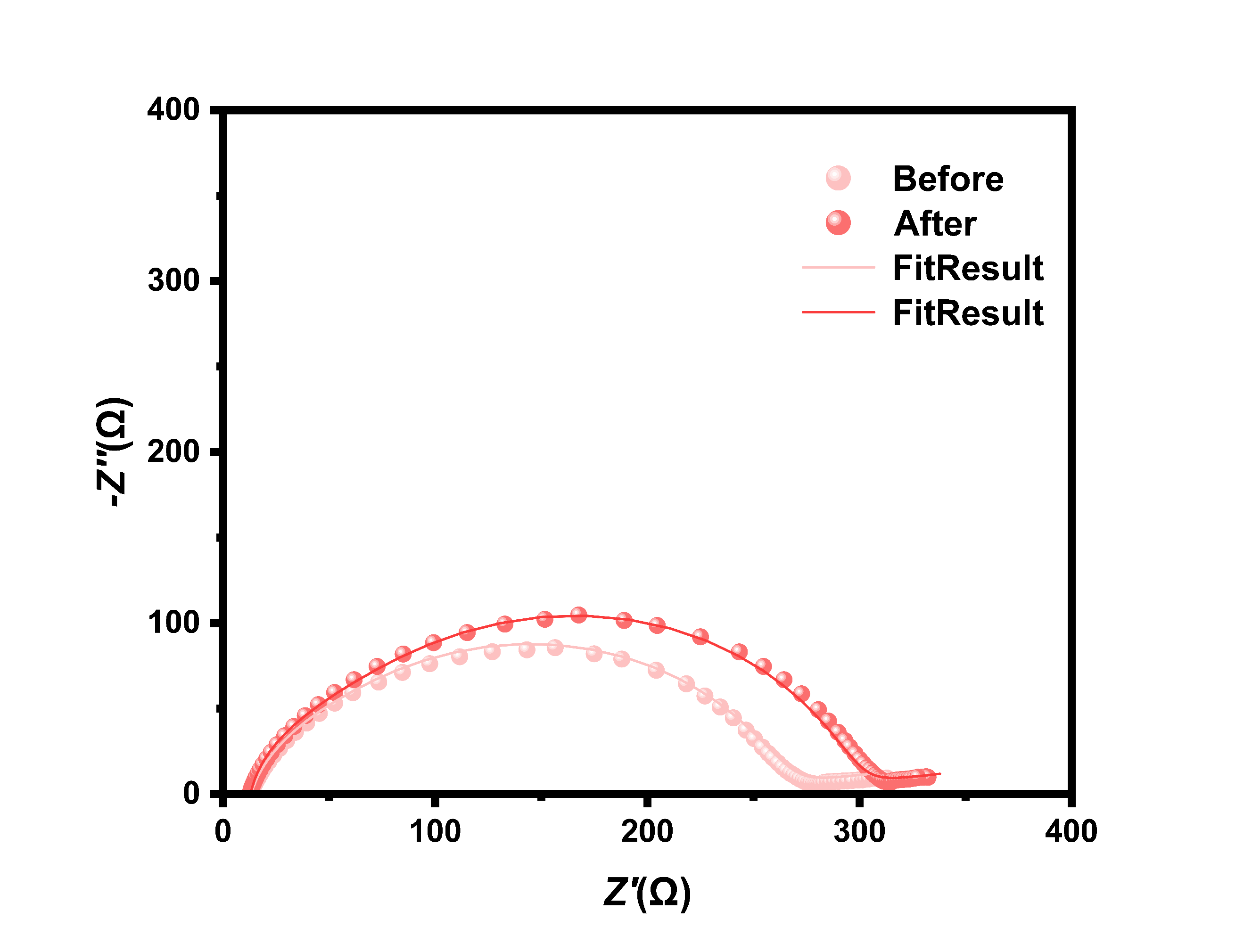


**Figure S9** Equivalent circuit for modelling EIS plots of symmetric Li||Li batteries (Take PMBA-PPR_5_ as an example).

The impedance spectra were fitted using an equivalent circuit consisting of a bulk resistance (R_1_), two parallel R–CPE elements (R_2_–CPE_1_ and R_3_–CPE_2_), and a finite-length Warburg element (W_o1_). R_1_ represents the bulk resistance of the electrolyte, while R_2_ and CPE_1_ account for the interphase resistance and non-ideal capacitive behavior of the SEI or polymer/Li interface. R_3_ and CPE_2_ correspond to the charge-transfer resistance and associated double-layer capacitance at the electrode/electrolyte interface. The W_o1_ element captures lithium-ion diffusion through the interfacial layer and into the electrolyte. This model provides a more accurate fit and better resolution of electrochemical processes.


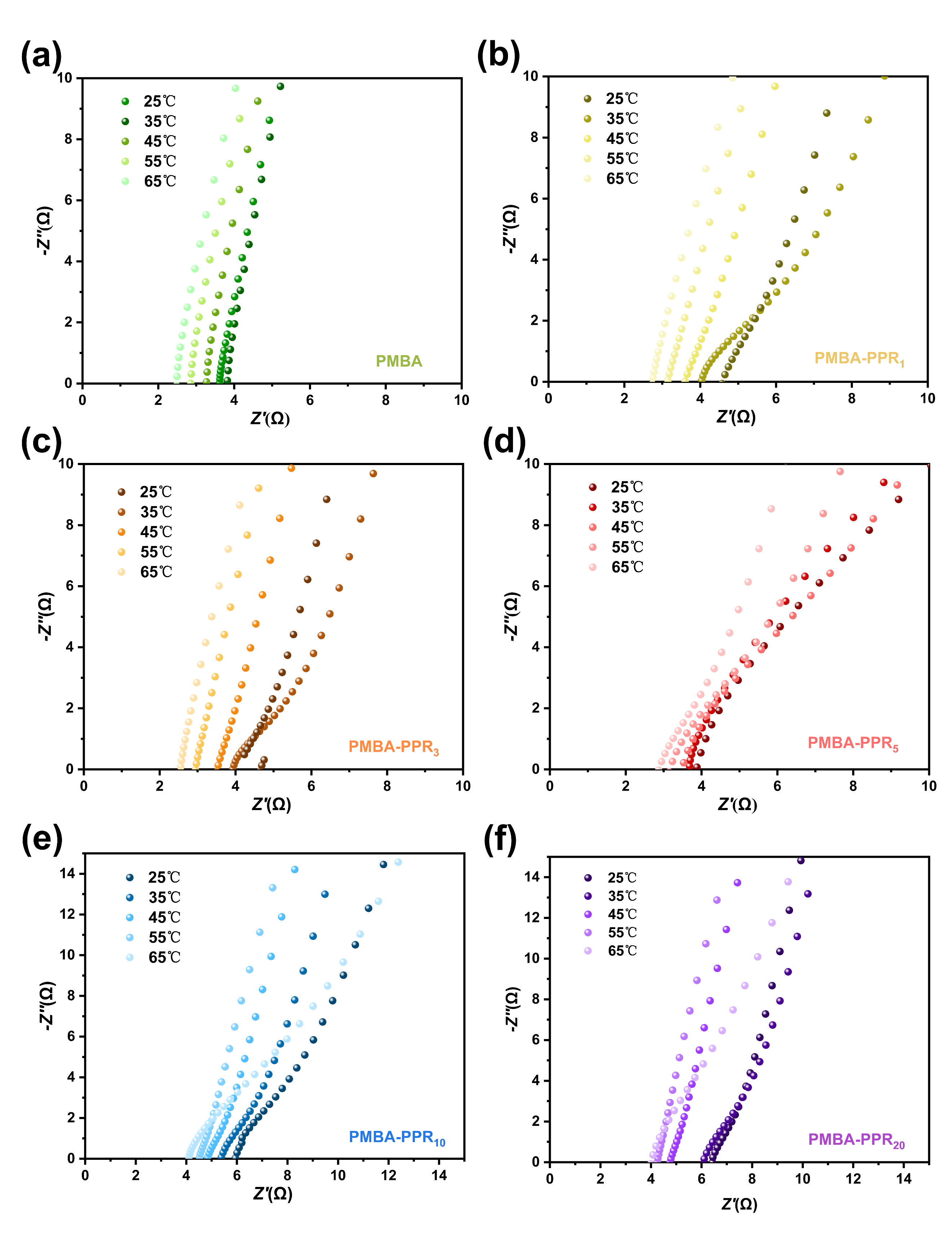


**Figure S10** EIS spectra of electrolytes with temperature change. (a)PMBA. (b)PMBA-PPR_1_. (c)PMBA-PPR_3_. (d)PMBA-PPR_5_. (e)PMBA-PPR_10_. (f)PMBA-PPR_20._


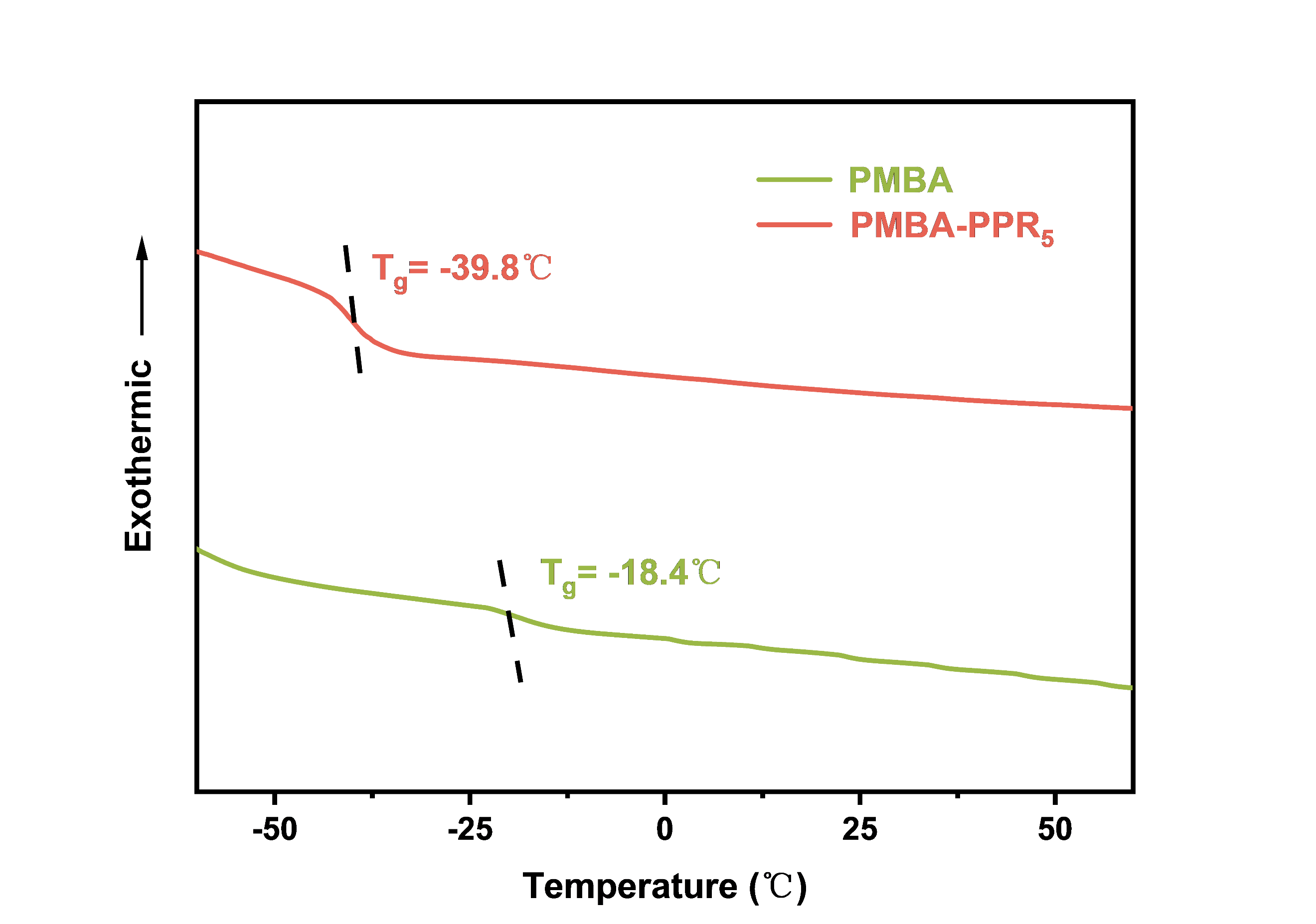


**Figure S11** DSC curves of PMBA and PMBA-PPR_5_.


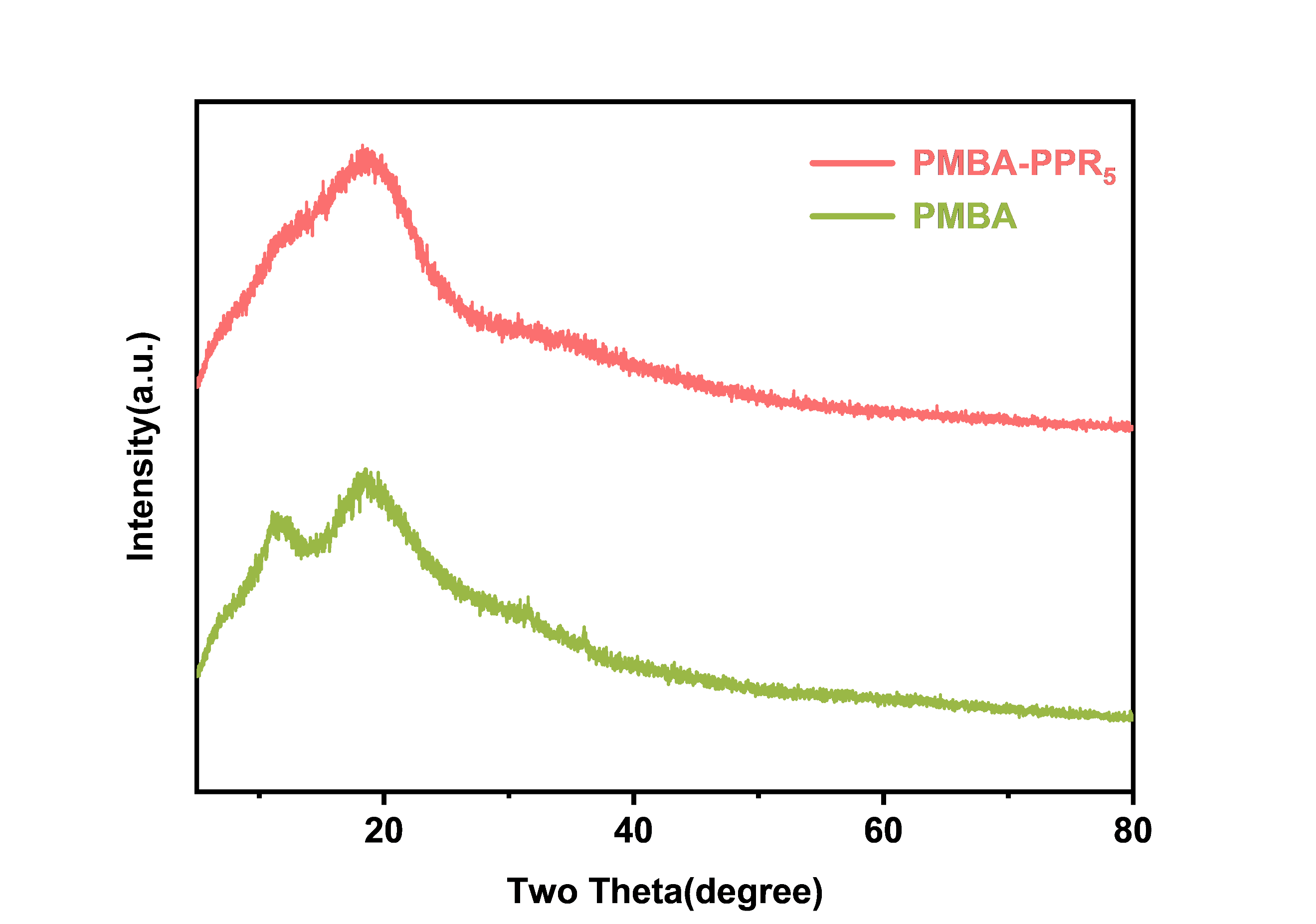


**Figure S12** XRD image of PMBA and PMBA-PPR_5_.


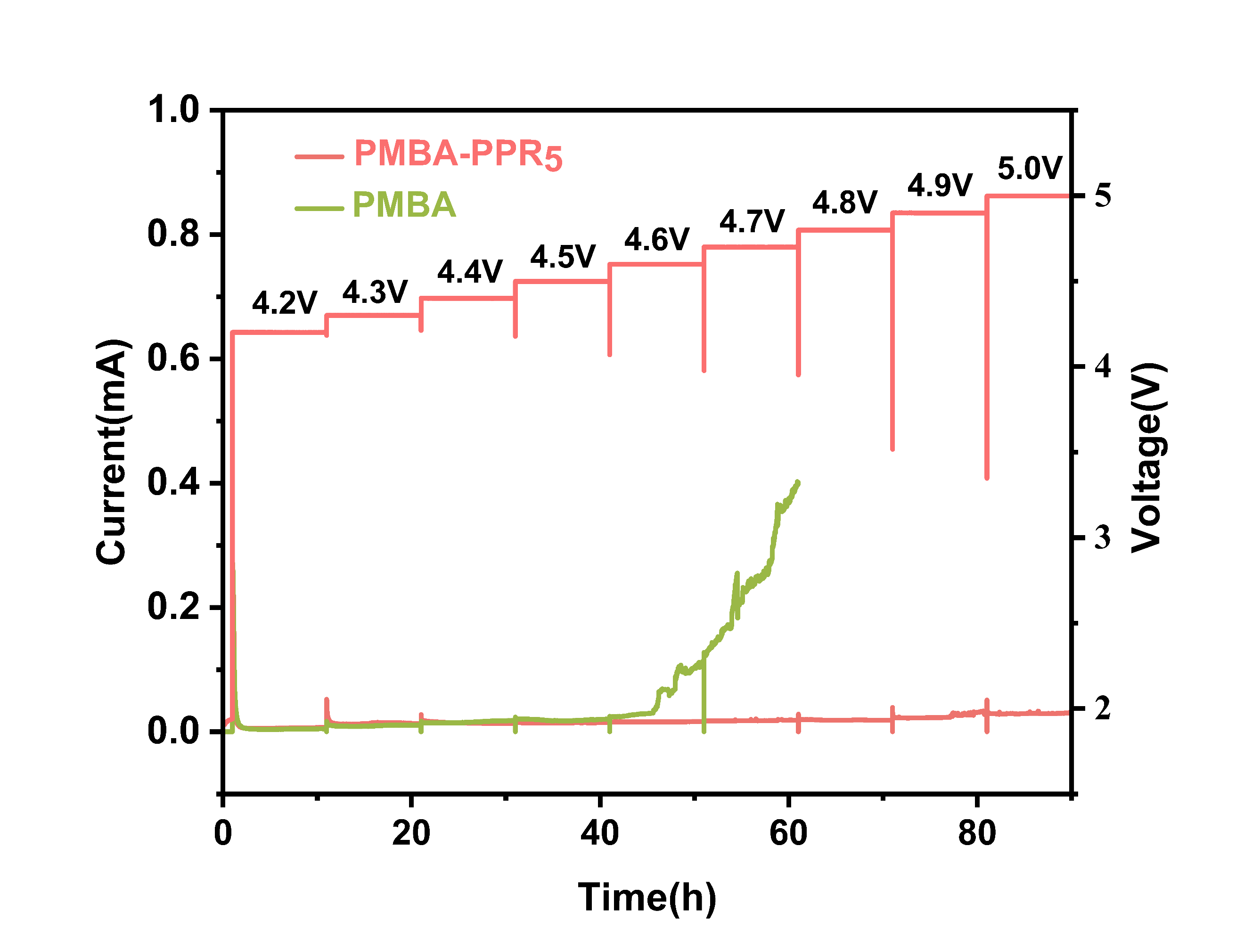


**Figure S13** Electrochemical floating curves of NCM811||Li cells containing PMBA and PMBA-PPR_5_ electrolytes.


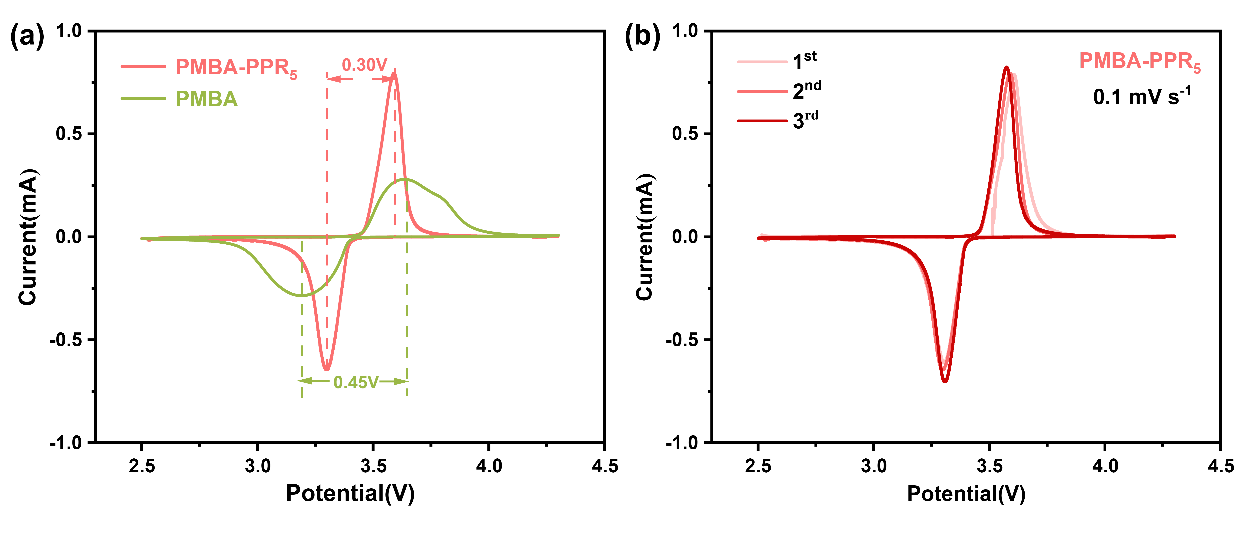


**Figure S14** (a) CV curves of Li|PMBA|LFP and Li|PMBA-PPR_5_|LFP at 0.1 mV s^-1^ scanning rate. (b) Li| PMBA-PPR_5_|LFP CV curve repeated three times at 0.1 mV s^-1^ scanning rate.


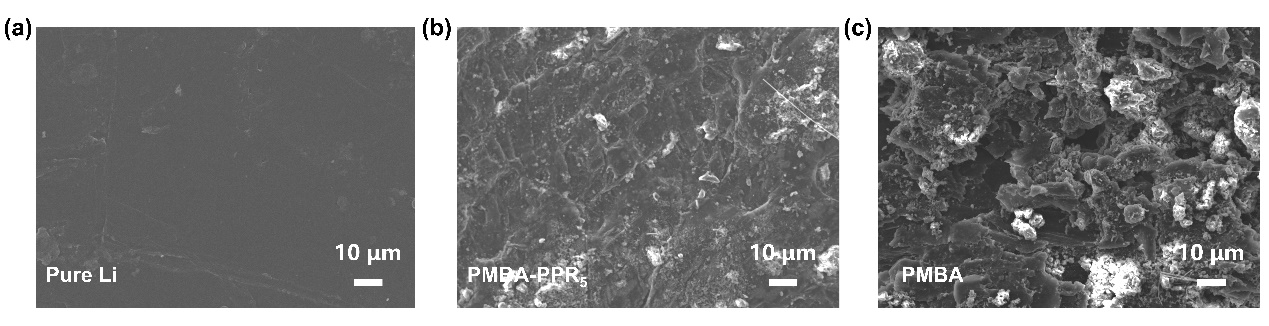


**Figure S15** SEM images of Li metal surface. (a) Pure Li before cycling. (b) Li metal of Li| PMBA-PPR_5_|Li cell after cycling 2000 h. (c) Li metal of Li|PMBA|Li cell after cycling 2000 h.


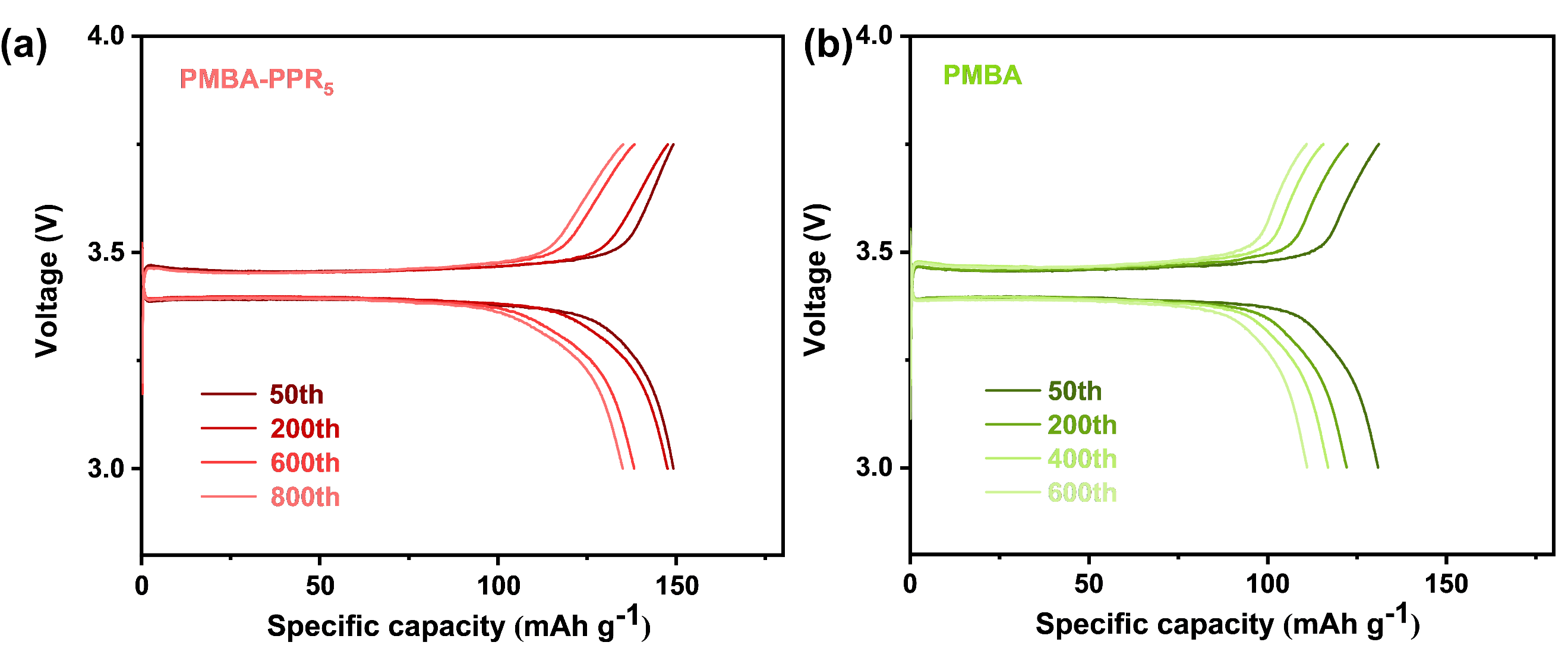


**Figure S16** Capacity-voltage curves of electrolytes at different cycles with low active mass loading LFP cathodes. (a) Li|PMBA-PPR_5_|LFP. (b) Li|PMBA|LFP.


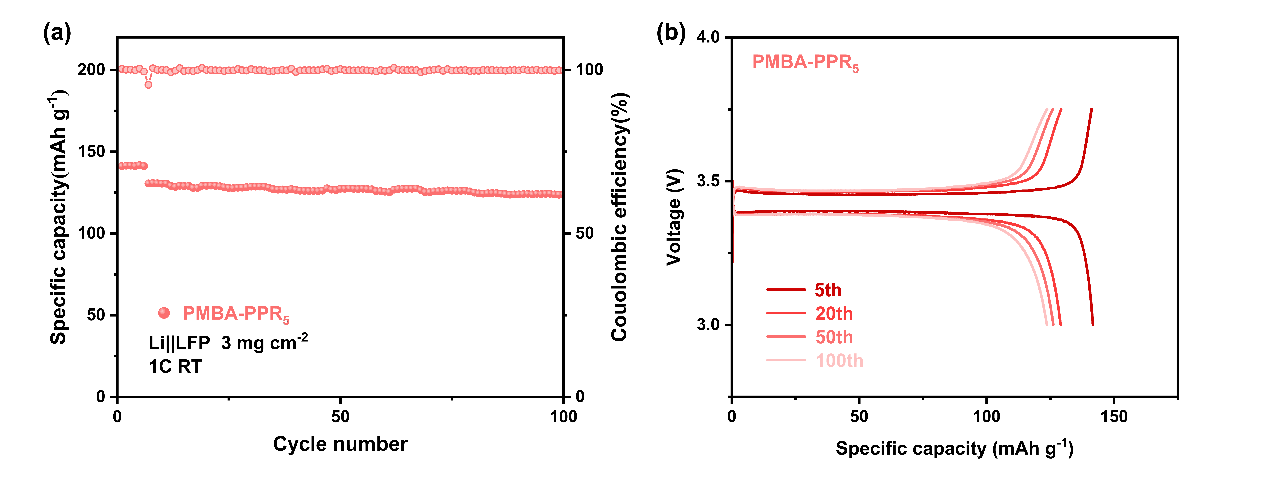


**Figure S17** Li|PMBA-PPR_5_|LFP cell at 1C (a) cycle performance. (b) Capacity-voltage curves of PMBA-PPR_5_.


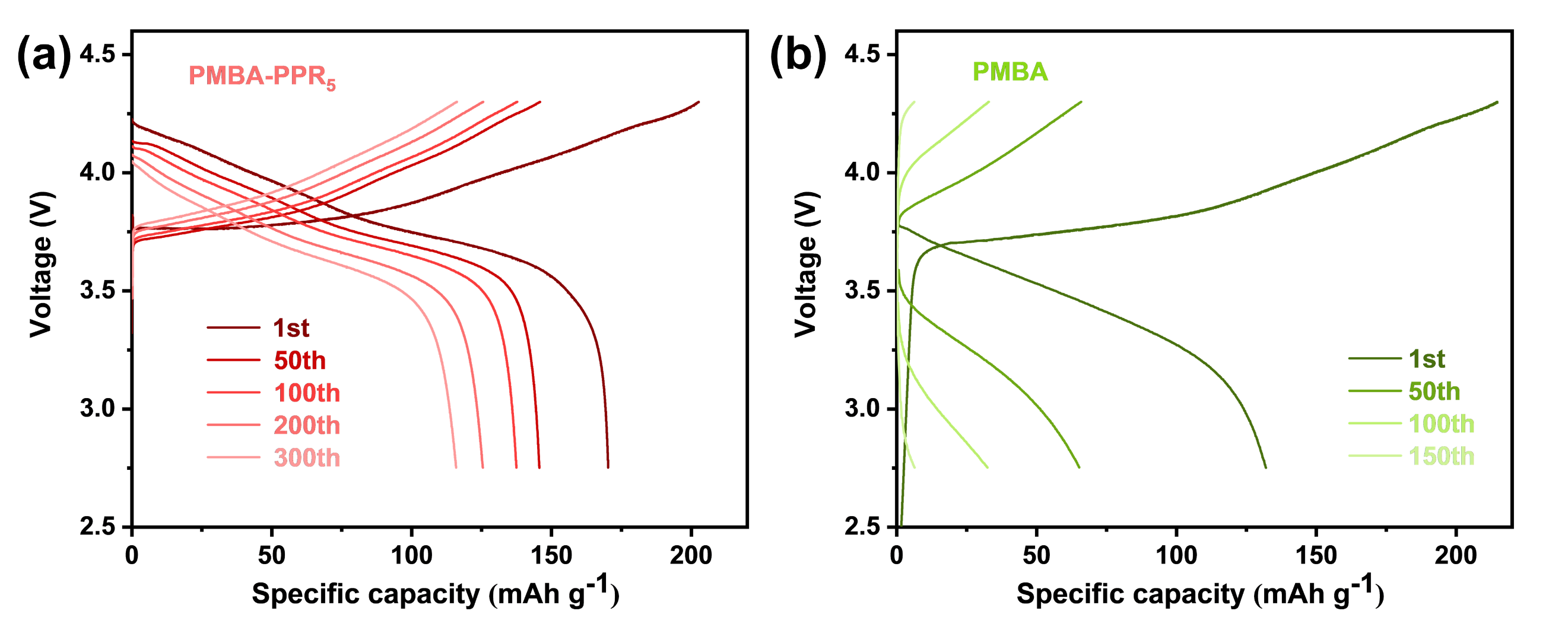


**Figure S18** Capacity-voltage curves of electrolytes at different cycles. (a) Li| PMBA-PPR_5_|NCM811. (b) Li|PMBA|NCM811.


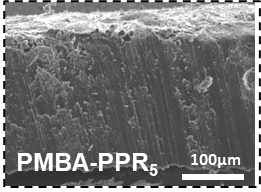

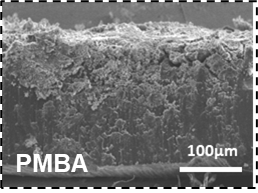


**Figure S19** The cross-section SEM images of Li anode surface from Li||NCM811 cells after 100 cycles.


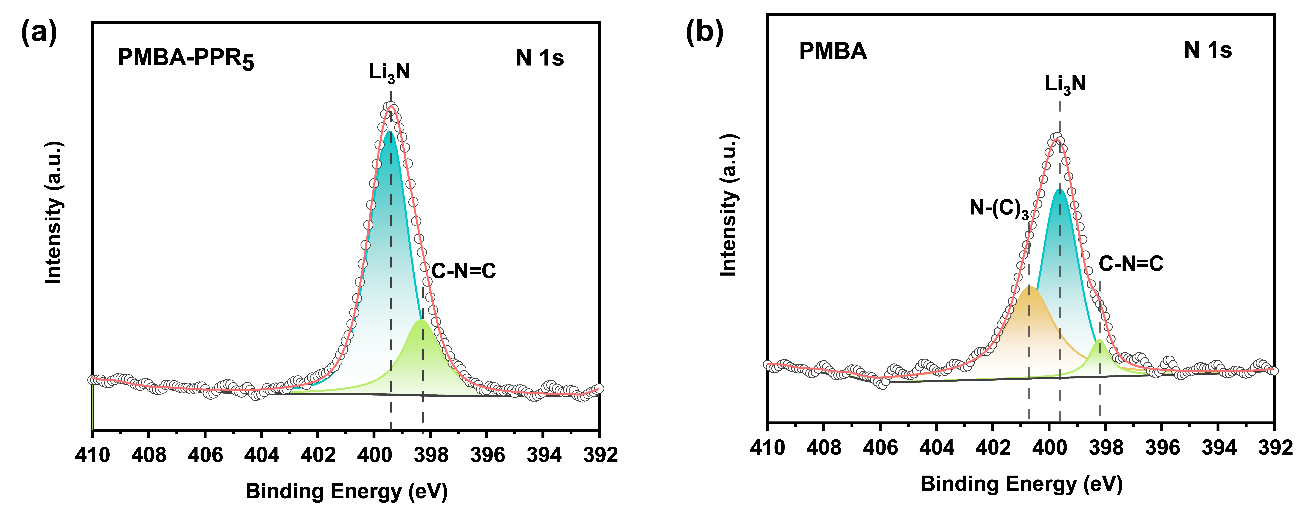


**Figure S20** XPS spectrum of lithium metal surface after 100 cycles: PMBA-PPR_5_ (a) N1s. PMBA (b) N 1s.


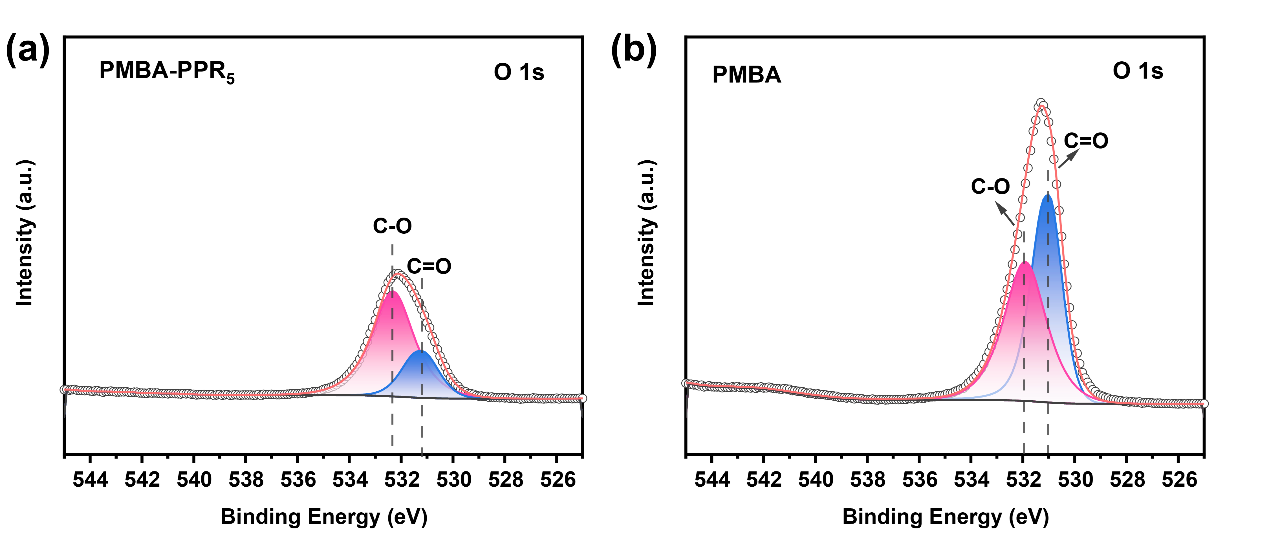


**Figure S21** XPS spectrum of NCM811 cathodes after 100 cycles: PMBA-PPR_5_ (a) O 1s. PMBA (b) O 1s.

**Table S2** Comparison of electrolyte properties reported in relevant literatures

| Electrolyte | Working Temperature [℃] | Cathode | Rate[C] | Cycle number | Capacity retention [%] | Ref |
| --- | --- | --- | --- | --- | --- | --- |
| SIGE | 25 | LFP | 0.2 | 200 | 98 | [1] |
| SCGPE-3 | 30 | LFP | 1 | 1000 | 89.6 | [2] |
|  | 30 | NCM811 | 0.5 | 150 | 70 |  |
| PRX-SPE | 60 | LFP | 1 | 100 | 98.5 | [3] |
| PR-PAA | 25 | NCM622 | 0.5 | 150 | 80 | [4] |
| CPE | 25 | LFP | 0.5 | 250 | 96.5 | [5] |
|  | 25 | NCA | 0.1 | 100 | 84.4 |  |
| GPE | 25 | LLO | 1 | 500 | 86.7 | [6] |
| PMBA-PPR_5_ | 25 | LFP | 0.5 | 1000 | 90.2 | This work |
|  | 25 | NCM811 | 0.5 | 300 | 85 |  |

**Reference**

[1] W. Ma, H. Wu, Y. Cai, Z. Yu, Y. Wang, J.-H. Zhang, Q. Zhang, X. Jia, A flexible single-ion gel electrolyte with a multiscale channel for the high-performance lithium metal batteries, *ACS Materials Lett.* 4 (5) (2022) 944–952.

https://doi.org/10.1021/acsmaterialslett.2c00188.

[2] X. Deng, J. Chen, X. Jia, X. Da, Y. Zhao, Y. Gao, Y. Gao, X. Kong, S. Ding, G. Gao, Highly tough slide‐crosslinked gel polymer electrolyte for stable lithium metal batteries, *Angew Chem Int Ed* (2024) e202410818.

https://doi.org/10.1002/anie.202410818.

[3] J. Seo, G. Lee, J. Hur, M. Sung, J. Seo, D. Kim, Mechanically interlocked polymer electrolyte with built‐in fast molecular shuttles for all‐solid‐state lithium batteries, *Advanced Energy Materials* 11 (44) (2021) 2102583.

https://doi.org/10.1002/aenm.202102583.

[4] R. Gao, H. Yang, C. Wang, H. Ye, F. Cao, Z. Guo, Fatigue‐resistant interfacial layer for safe lithium metal batteries, *Angew Chem Int Ed* 60 (48) (2021) 25508–25513. https://doi.org/10.1002/anie.202111199.

[5] P. Ding, L. Wu, Z. Lin, C. Lou, M. Tang, X. Guo, H. Guo, Y. Wang, H. Yu, Molecular Self-Assembled Ether-Based Polyrotaxane Solid Electrolyte for Lithium Metal Batteries, *J. Am. Chem. Soc.* 145 (3) (2023) 1548–1556.

https://doi.org/10.1021/jacs.2c06512.

[6] C. Zhang, Z. Lu, M. Song, Y. Zhang, C. Jing, L. Chen, X. Ji, W. Wei, Highly Oxidation‐Resistant Ether Gel Electrolytes for 4.7 V High‐Safety Lithium Metal Batteries, *Advanced Energy Materials* 13 (21) (2023) 2203870.

https://doi.org/10.1002/aenm.202203870.
